# The PGD_2_/DP2 axis promotes pro-inflammatory responses but attenuates an antigen-presenting role of human basophils

**DOI:** 10.1038/s42003-026-10286-w

**Published:** 2026-05-20

**Authors:** Jian Luo, Wentao Chen, Martin J. Lett, Simin Yu, Wei Luo, Emma Coakley, Shamsah Kazani, Eric A. Shikatani, Timothy S. C. Hinks, David A. Sandham, Ian D. Pavord, Paul Klenerman, Luzheng Xue

**Affiliations:** 1 Respiratory Medicine Unit, and Oxford NIHR Biomedical Research Centre, University of Oxford, Oxford, UK; 2 Translational Gastroenterology Unit and Peter Medawar Building, University of Oxford, Oxford, UK; 3 Novartis Biomedical Research, Cambridge, MA USA

**Keywords:** Immunology, Inflammation, Asthma

## Abstract

Basophils are distinct blood leucocytes that express immunoglobulin E (IgE) receptor FcεR1 and can be activated by IgE. Their roles in asthma, particularly IgE-independent effects, remain incompletely understood. Human basophils highly express the prostaglandin D_2_ (PGD_2_) receptor 2 (DP2; also known as chemoattractant receptor homologous molecule expressed on Th2 cells, CRTH2). Here, we explore the PGD_2_/DP2 axis as an alternative pathway for basophil activation. Basophils are enriched in patients with severe eosinophilic asthma, correlating with eosinophil counts but not IgE levels. PGD_2_/DP2 stimulation promotes basophil recruitment, activation, and the production of histamine, leukotrienes, and type 2 cytokines. RNA sequencing and further in vitro experiments reveal that, although PGD_2_ and IgE share some signalling pathways and functions, they induce distinct gene transcriptional signatures. Interestingly, PGD_2_ downregulates major histocompatibility complex class I-related gene protein (MR1) and attenuates basophil antigen-presentation to mucosal-associated invariant T (MAIT) cells, leading to reduced IFN-γ production. These findings highlight the PGD_2_/DP2/basophil axis as a contributor to asthma, particularly in IgE-low conditions, and suggest it as a potential therapeutic target for related diseases.

## Abstract (web summary)

A previously unrecognized MR1-dependent antigen-presenting role for human basophils in MAIT-cell activation is identified; PGD_2_/DP2 signalling negatively regulates this pathway to suppress MAIT-derived IFN-γ production.

## Introduction

Basophils are a population of peripheral blood leucocytes containing cytoplasmic granules that stain with basophilic dyes. They have short lifespans, typically around 5 days or less in vivo^1^. Basophils are functionally distinct from other leucocytes due to the expression of high-affinity immunoglobulin (Ig) E receptor FcεR1 and their ability to be activated through IgE-mediated FcεR1 crosslinking. Because of their similarity to mast cells, basophils have often been considered redundant and negligible “circulating mast cells”. Activation of basophils leads to the rapid release of preformed histamine, newly synthesised leukotriene C_4_ (LTC_4_), and type 2 cytokines interleukin (IL)-4 and IL-13^2^. Both histamine and LTC_4_ are potent bronchoconstrictors^3,4^, contributing to acute asthma attacks through smooth muscle constriction. IL-4 and IL-13 are key cytokines in type 2 cell differentiation and airway epithelial activation, playing critical roles in asthma pathogenesis^5^. However, compared to other immune effector cells, the roles of basophils in asthma remain poorly understood. In particular, the potential roles of human basophils in antigen presentation and asthma exacerbation have not been explored, although antigen-presenting capacity has been observed in mouse basophils^6–8^.

In human circulating blood, all basophils express prostaglandin D_2_ receptor 2 (DP2; also known as chemoattractant receptor homologous molecule expressed on Th2 cells, CRTH2). The expression level of DP2 on basophils is one of the highest among all DP2^+^ immune cells, including type 2 T helpers (Th2), type 2 cytotoxic T cells (Tc2), group 2 innate lymphoid cells (ILC2) and eosinophils^9,10^. Prostaglandin D_2_ (PGD_2_) is the major arachidonic acid metabolite released from mast cells during an allergic response, although macrophages, dendritic cells, Th2, Tc2 and ILC2 cells may also contribute to PGD_2_ production in certain circumstances^11–15^. It is well-established that, through DP2, PGD_2_ elicits cell migration, pro-inflammatory cytokine production and suppression of apoptosis in Th2, Tc2 and ILC2 cells^10,16,17^. Although PGD_2_ has been shown to promote basophil migration and activation via DP2 in vitro^9,18^, the roles of the PGD_2_/DP2 axis in basophils are not yet fully understood. Basophil numbers are increased in the patients with asthma or other allergic disorders^19–21^, however, how PGD_2_/DP2-mediated basophil activation, an IgE/FcεR1-independent pathway, contributes to the pathogenesis of these diseases remain unclear.

In this study, we investigated the distribution profiles of basophils in blood and sputum in a well-defined patient cohort with various asthma phenotypes, as well as in healthy controls. We then explored the pathogenic roles of basophils via PGD_2_/DP2 axis in type-2 inflammation using the DP2 antagonist QAW039 (fevipiprant). To gain a broad understanding of basophil biology, we performed RNA sequencing (RNAseq) on purified blood basophils treated with different stimuli. We also further examined the signal mechanisms involved in the PGD_2_/DP2-mediated basophil activation, as well as a previously unknown role of PGD_2_/DP2 in basophil/major histocompatibility complex class I-related gene protein (MR1)-mediated antigen presentation. These findings will help expand our knowledge of basophil biology and their roles in disease, and could contribute to the development of basophil-based therapeutic strategies in related disorders.

## Results

### Basophils are enriched in patients with severe eosinophilic asthma

**Fig. 1:**
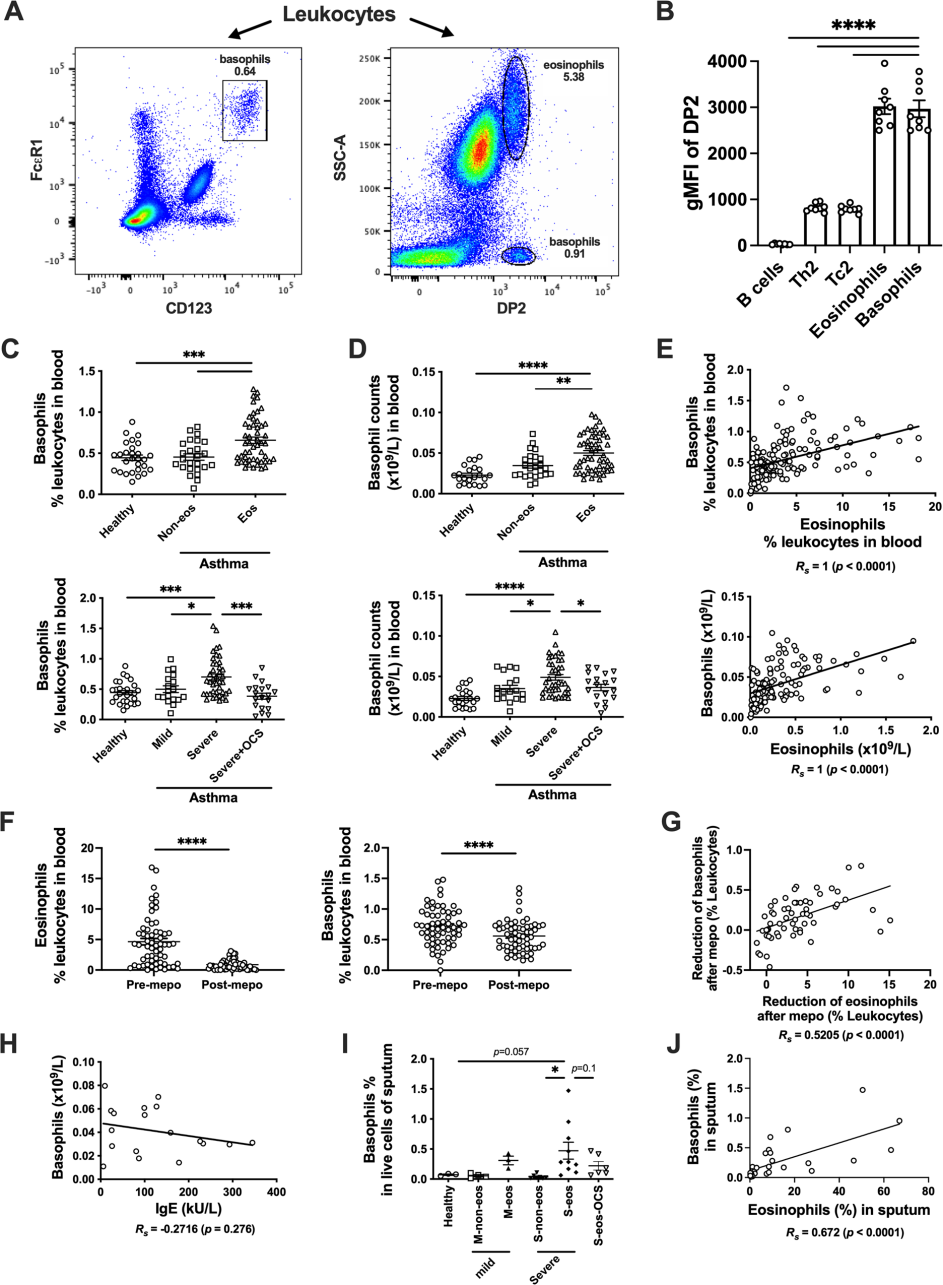
Basophils are enriched in patients with severe eosinophilic asthma. **A** CD123^+^FcεR1^+^DP2^high^SSC^low^ basophils express higher levels of FcεR1 and DP2 than most of other leucocytes determined by flow cytometry. **B** Levels of DP2 in basophils are similar to those in eosinophils and higher than those in other leucocytes, including B, Th2 and Tc2 cells detected by flow cytometry. *n* = 8. Frequencies of basophils in total leucocytes (**C**) or numbers of basophils (**D**) in peripheral blood were compared between healthy controls and asthma patient groups defined by eosinophil counts (non-eosinophilic and eosinophilic) or asthma severity (mild, severe and severe with OCS) with flow cytometry. *n* = 28 for healthy, *n* = 25 for non-eos, *n* = 51 for eos, *n* = 18 for mild, *n* = 41 for severe and *n* = 19 for severe/OCS. **E** Correlation of basophil frequencies or counts and eosinophil frequencies or counts in peripheral blood. *n* = 133. **F** Frequencies of blood eosinophils or basophils before or after mepolizumab treatment in paired patient samples. *n* = 60. **G** Correlation analysis of eosinophil downregulation and basophil downregulation after mepolizumab treatment. *n* = 60. **H** Correlation analysis of basophil counts and IgE levels in blood. *n* = 18. **I** Frequencies of basophils in total live cells in induced sputa were compared between healthy controls and asthma patient groups with flow cytometry. *n* = 3 for healthy and M-eos, *n* = 4 for M-non-eos, *n* = 7 for S-non-eos, *n* = 10 for S-eos and *n* = 6 for S-eos/OCS. **J** Correlation of basophil frequencies and eosinophil frequencies in sputum. *n* = 30. *p < 0.05, **p < 0.01, ***p < 0.001, ****p < 0.0001.

The expression levels of FcεR1, CD123 and DP2 are specifically high in human blood basophils compared to most of other leucocytes (Fig. 1A). The DP2 level in basophils is similar to that in eosinophils (Fig. 1B). We used SSC^low^CD193^+^CD123^+^DP2^high^ to define basophils in our flow cytometry analysis to study the basophil profiles in human asthma phenotypes (Supplementary Fig. 1). In a cohort from Oxford, UK, peripheral blood basophils were significantly higher in patients with eosinophilic (~0.66 ± 0.28% of leucocytes, *n* = 51) or severe (~0.70 ± 0.32%, *n* = 41) asthma compared to those with non-eosinophilic (~0.45 ± 0.19%, *n* = 25) or mild (~0.50 ± 0.24%, *n* = 18) asthma or healthy controls (~0.44 ± 0.18%, *n* = 28; Fig. 1C; Supplementary Table 1). Oral corticosteroid (OCS) treatment significantly reduced blood basophil levels in patients with severe eosinophilic asthma (~0.39 ± 0.22%, *n* = 19). Findings were similar when blood basophils were expressed as absolute cell numbers (Fig. 1D). Peripheral blood basophil frequencies or counts were positively correlated with eosinophil (SSC^high^CD193^+^CD123^+^DP2^high^) frequencies or counts (Spearman r (*Rs*) = 1, *p* < 0.0001; Fig. 1E and Supplementary Fig. 1). In patients with severe eosinophilic asthma treated with mepolizumab, an anti-IL-5 therapy, both eosinophil and basophil frequencies were suppressed after the treatment (Fig. 1F; Supplementary Table 1). Although basophil suppression was not as pronounced as eosinophil reduction, the two were highly correlated (*Rs* = 0.52, *p* < 0.0001; Fig. 1G and Supplementary Fig. 2). However, blood basophil counts showed no association with IgE levels in the blood (*Rs* = -0.27, *p* = 0.28; Fig. 1H).

Similar to peripheral blood, basophil levels were also elevated in sputum samples from patients with eosinophilic asthma (~ 0.48 ± 0.44% in live cells, *n* = 10) compared to those with non-eosinophilic asthma (~0.051 ± 0.04%, *n* = 7) or healthy individuals (~0.071 ± 0.018%, *n* = 3; Fig. 1I). OCS treatment reduced sputum basophils in patients with severe eosinophilic asthma (~0.22 ± 0.17%, *n* = 6). Basophil levels in sputum were positively correlated with eosinophil levels (*Rs* = 0.67, *p* < 0.0001; Fig. 1J).

### PGD_2_/DP2 axis induces basophil adhesion and migration

**Fig. 2:**
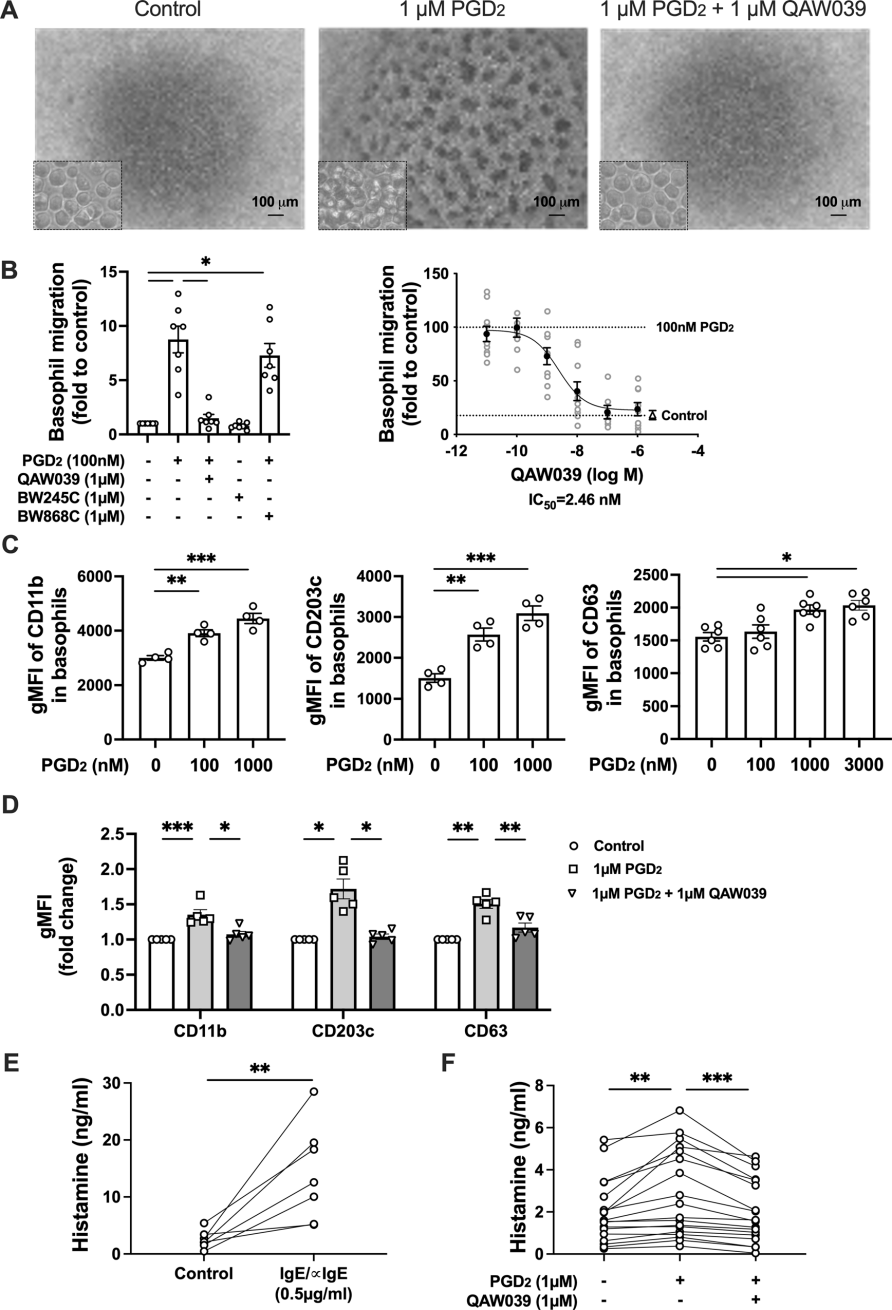
PGD_2_-induced cell migration and activation of basophils were inhibited by DP2 antagonist. **A** Representative images of cell aggregation of isolated basophils after incubation with 1 µM PGD_2_ for 1.5 h was reversed by 1 µM QAW039, as recorded under microscopy. **B** Basophil migration induced by 100 nM PGD_2_ in the presence or absence of 1 µM BW245C (DP1 agonist), 1 µM BW868C (DP1 antagonist), or various concentrations of QAW039 as determined by chemotaxis assay. *n* = 7 for left panel and *n* = 10 for right panel. **C** and **D** Levels of cell activation markers CD11b, CD203c and CD63 on the surface of basophils in fresh blood after incubation with different concentrations of PGD_2_ **C**, or with 1 µM PGD_2_ in the absence or presence of 1 µM QAW039 **D** for 30 min were measured with flow cytometry. *n* = 4 for left and middle panels, and *n* = 6 for right panel in (**C**); *n* = 5 for **D**. Levels of histamine in the supernatants of isolated basophils after treatment with 0.5 µg/ml IgE/anti-IgE (**E**), or 1 µM PGD_2_ in the absence or presence of 1 µM QAW039 (**F**) for 0.5 h measured with ELISA. *n* = 7 for **E** and *n* = 18 for **F**. *p < 0.05, **p < 0.01, ***p < 0.001.

Since both basophils and PGD_2_ are enriched in the airways of patients with severe eosinophilic asthma (Fig. 1I)^17,21^, to understand the potential roles of human basophils in asthma pathogenesis, we isolated basophils from fresh blood samples (Supplementary Fig. 3) and examined their responses to PGD_2_ in processes related to cell recruitment, including adhesion and migration.

in vitro aggregation of purified basophils is a marker of basophil activation and adhesion^22^. Significant aggregation of basophils occurred rapidly within 0.5 h after PGD_2_ (1 µM) treatment and persisted for over 2 h (Fig. 2A). This effect was mediated by DP2, as DP2 is the dominant PGD_2_ receptor in basophils (Supplementary Fig. 4), and the DP2-specific antagonist QAW039 (1 µM) completely inhibited aggregation (Fig. 2A).

PGD_2_ (100 nM) also strongly promoted basophil migration in a chemotaxis assay (Fig. 2B). Migration was specifically mediated by DP2, as the DP1 agonist BW245C did not induce migration, and the DP1 antagonist BW868C did not inhibit migration. In contrast, QAW039 inhibited migration in a dose-dependent manner with an IC_50_ of 2.46 nM.

The adhesion molecule, CD11b, required for basophil aggregation and migration, was significantly upregulated by PGD_2_, which was inhibited by QAW039 (Fig. 2C, D).

### PGD_2_/DP2 axis induces basophil activation

Next, we examined the effect of PGD_2_/DP2 signalling on general basophil activation using activation markers (Fig. 2C, D). The levels of CD11b (an adhesion molecule), CD203c (an activation marker), and CD63 (a degranulation marker) on the basophil surface were upregulated in PGD_2_-treated fresh blood in a concentration-dependent manner (Fig. 2C and Supplementary Fig. 5A). Notably, CD63 upregulation was less pronounced than that of CD11b and CD203c. These upregulations were inhibited by QAW039 (1 µM) (Fig. 2D).

Histamine release is a key feature of basophil activation^2^. FcεR1 stimulation with IgE/anti-IgE induced significant histamine release in basophils (Fig. 2E). Histamine release was also detectable after PGD_2_ treatment for ≥30 min, although to a lesser extent than IgE/anti-IgE stimulation, and this response was inhibited by QAW039 (1 µM) (Fig. 2F and Supplementary Fig. 5B).

### PGD_2_/DP2 axis induces type-2 cytokine production by basophils

**Fig. 3:**
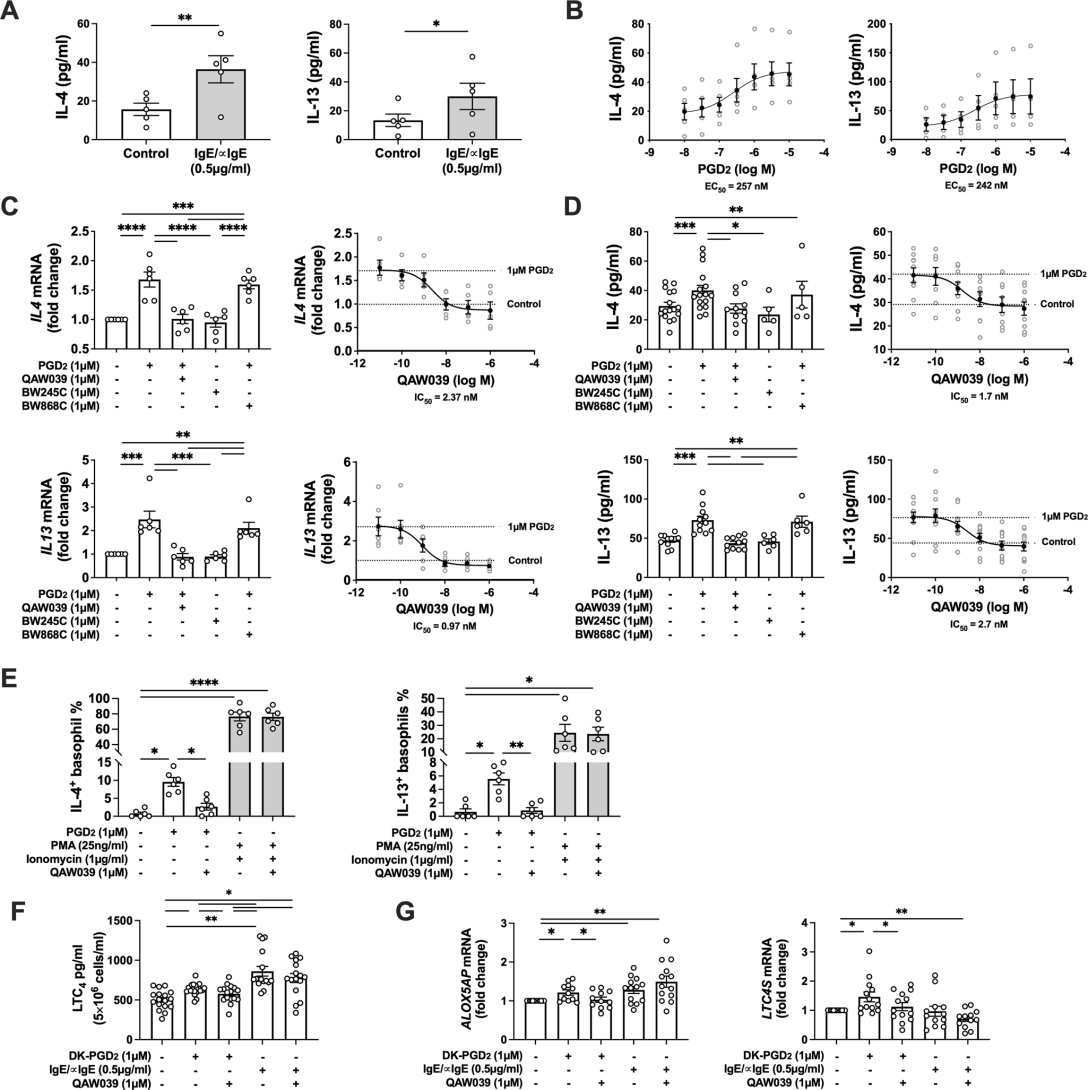
DP2-mediated type 2 cytokine and leukotriene C_4_ (LTC_4_) production in basophils was blocked by DP2 antagonist in vitro and ex vivo. **A** Levels of IL-4 and IL-13 released by isolated basophils after treatments with 0.5 µg/ml IgE/anti-IgE were detected with ELISA. *n* = 5. **B** Release of IL-4 and IL-13 by isolated basophils in response to various concentrations of PGD_2_. *n* = 5. mRNA (**C**) or protein (**D**) levels for IL-4 and IL-13 in isolated basophils after treatments with 1 µM PGD_2_ in the presence or absence of BW245C, BW868C, or various concentrations of QAW039 as measured with qRT-PCR or ELISA, respectively. *n* = 6 for **C**; *n* = 16 for control or PGD_2_, *n* = 11 for PGD_2_/QAW039, *n* = 5 for BW245C or BW245C/BW868C in top left panels of **D**; *n* = 11 for control, PGD_2_ or PGD_2_/QAW039, *n* = 6 for BW245C or BW245C/BW868C in bottom left panels of **D**; *n* = 9 for top right panel of **D**, and *n* = 11 for bottom right panel of **D**. **E** IL-4- and IL-13-producing basophils induced by PGD_2_ (1 µM) or PMA (25 ng/ml)/ionomycin (1 µg/ml) in the absence or presence of QAW039 (1 µM) in fresh blood from severe asthmatics as detected by using intracellular staining for flow cytometry. *n* = 6. Levels of LTC_4_ in the supernatants of isolated basophils (**F**) and mRNA levels of *ALOX5AP* (gene for 5-lipoxygenase activating protein) and *LTC4S* (gene for leukotriene C4 synthase) in basophils (**G**) after treatments with DK-PGD_2_ or IgE/anti-IgE in the presence or absence of QAW039, detected with ELISA or qPCR respectively. *n* = 21 for **F** and *n* = 13 for **G**. *p < 0.05, **p < 0.01, ***p < 0.001, ****p < 0.0001.

Basophils have been reported to produce type-2 cytokines such as IL-4 and IL-13 in response to IgE-mediated FcεR1 crosslinking^23^, which was confirmed in vitro using isolated human basophils (Fig. 3A). IL-5 production was not detectable in basophils. PGD_2_ also stimulated IL-4/13 productions in a dose-dependent manner with EC_50_ = 257 nM for IL-4 and EC_50_ = 242 nM for IL-13 (Fig. 3B). To further confirm the receptor mediating PGD_2_-induced cytokine production, basophils were incubated with PGD_2_ (1 µM) or BW245C (1 µM) in the presence or absence of BW868C (1 µM) or various concentrations of QAW039 (Fig. 3C, D). IL-4 and IL-13 production was significantly enhanced at both transcriptional (Fig. 3C) and protein levels (Fig. 3D) in response to PGD_2_, specifically mediated by DP2, as the effect was inhibited by QAW039 but not by BW868C. The inhibitory effect of QAW039 was dose-dependent, with IC_50_ values of 2.37 nM for *IL4* and 0.97 nM for *IL13* at mRNA levels, and 1.7 nM for IL-4 and 2.7 nM for IL-13 at protein levels.

The physiological relevance of PGD_2_/DP2-stimulated cytokine production in basophils was confirmed by ex vivo intracellular staining of basophils from patients with severe asthma (Fig. 3E). The frequencies of IL-4^+^ or IL-13^+^ basophils increased in blood treated with PGD_2_ (1 µM) or phorbol 12-myristate 13-acetate (PMA, 25 ng/ml)/ionomycin (1 µg/ml). QAW039 (1 µM) completely inhibited PGD_2_-induced increase but not PMA/ionomycin-induced changes.

### PGD_2_/DP2 axis enhances LTC_4_ production and its biosynthetic pathway in basophils

To investigate the effect of PGD_2_ on the production of pro-inflammatory lipid mediators in basophils, we measured the concentrations of leukotrienes and PGD_2_ in the supernatants of basophils treated with DK-PGD_2_ or IgE/anti-IgE (Fig. 3F and Supplementary Fig. 5C). To differentiate between stimulator and product PGD_2_ in the PGD_2_ ELISA, we used DK-PGD_2_, a specific DP2 agonist that was undetectable in the assay, instead of PGD_2_ to stimulate cells. Both DK-PGD_2_ and IgE/anti-IgE stimulated basophils to release LTC_4_ (Fig. 3F) but not PGD_2_ (Supplementary Fig. 5C). The major leukotriene production from basophils was likely to be LTC_4_, as no significant change of LTE_4_ (Supplementary Fig. 5D) was detected. DK-PGD_2_-enhanced LTC_4_ production was inhibited by QAW039 (Fig. 3F).

To further understand the effect of PGD_2_ on LTC_4_ biosynthesis, we examined the expression of *ALOX5* (encoding 5-lipoxygenase, 5-LO), *ALOX5AP* (encoding 5-lipoxygenase-activating protein, FLAP) and *LTC4S* (encoding leukotriene C4 synthase, LTC4S), key enzymes required for leukotriene synthesis, using quantitative RT-PCR (qPCR, Fig. 3G and Supplementary Fig. 5E). *ALOX5* expression was unchanged by either DK-PGD_2_ or IgE/anti-IgE, whereas *ALOX5AP* was upregulated, particularly by IgE/anti-IgE. DK-PGD_2_ increased *LTC4S* expression, an effect reversed by QAW039. Expression of upstream enzymes phospholipase C (*PLC*) and phospholipase A2 (*PLA2*), remained unaffected (Supplementary Fig. 5F).

### PGD_2_/DP2 axis induces transcriptional changes in basophils

**Fig. 4:**
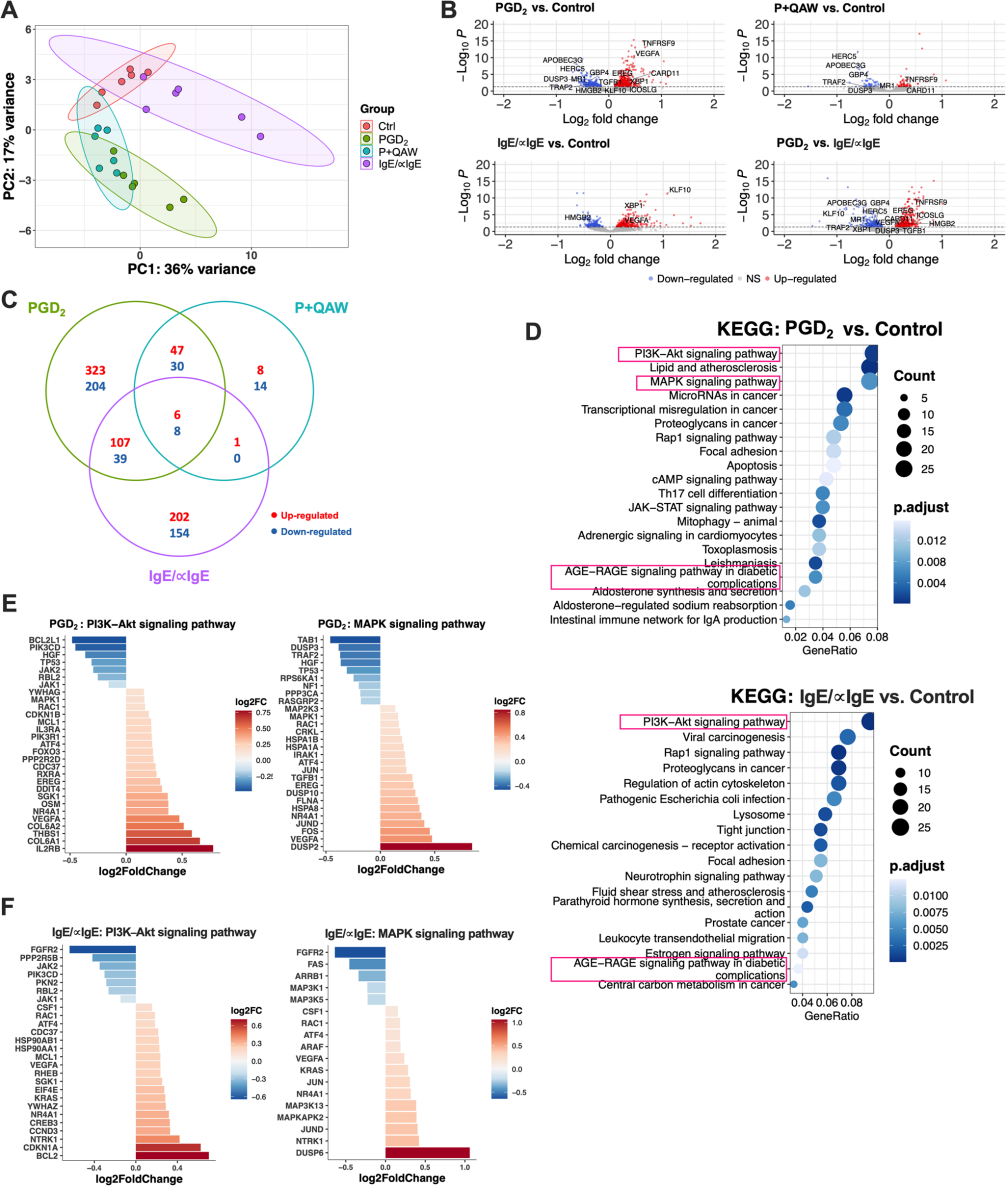
Effect of PGD_2_ and IgE on the transcriptional profiles of basophils detected by RNAseq. **A** PCA visualised the transcriptional distribution of basophils before or after treatment with 0.5 µg/ml IgE/anti-IgE or 1 µM PGD_2_ in the presence or absence of 1 mM QAW039. **B** Differential gene expression comparison between control, IgE/anti-IgE-treated, PGD_2_-treated and PGD_2_/QAW039-treated basophils. DEGs were defined as adjusted *p* < 0.05. **C** Venn diagram representing the numbers of DEGs regulated differently by IgE, PGD_2_ and PGD_2_/QAW039 in basophils. **D** Top 20 KEGG signalling pathways enriched in comparison between control and PGD_2_ or IgE/anti-IgE. Key genes involved in PI3K-Akt and MAPK signalling pathways in PGD_2_ (**E**) or IgE/anti-IgE stimulation (**F**).

To broadly investigate the roles of PGD_2_/DP2 axis and its differences from IgE-dependent activation in basophils, bulk-RNAseq was performed on basophils stimulated with PGD_2_ or IgE/anti-IgE. Principal component analysis (PCA) revealed distinct but partially overlapping transcription profiles between PGD_2_ and IgE/anti-IgE stimulation (Fig. 4A). Compared with control, PGD_2_ induced 764 differentially expressed genes (DEGs) and IgE/anti-IgE induced 517 DEGs, and the Log2 fold change of most genes were in the range of -1 and 1 (Fig. 4B). Among these, 160 DEGs (113 upregulated vs. 47 downregulated) were shared between PGD_2_ and IgE/anti-IgE (Fig. 4C). QAW039 blocked more than 88% of the gene changes induced by PGD_2_ (Fig. 4C). We also observed downregulation of *PTGDR2* (encoding DP2) following PGD_2_ treatment, which was partially reversed by QAW039 (Supplementary Fig. 6), consistent with findings in other cell types^10^, and indicative of basophil activation by PGD_2_.

Pathway analyses identified 217 Gene Ontology (GO) terms and 300 Kyoto Encyclopaedia of Genes and Genomes (KEGG) pathways enriched by PGD_2_ stimulation, while IgE/anti-IgE stimulation was associated with 55 GO terms and 285 KEGG pathways. Although PGD_2_ and IgE/anti-IgE showed different biological functions, both stimulators shared some similar functions, including inducing cell activation and migration, promoting cell survival, and responding to metabolites and cytokines (Supplementary Fig. 7). They also triggered several shared signalling pathways, including PI3K, MAPK and AGE-RAGE signalling (Fig. 4D). The gene sets involved in these signalling pathways are shown for PGD_2_ (Fig. 4E) and IgE/anti-IgE stimulation (Fig. 4F), with several shared genes such as *VEGFA*, *SK1*, *JAK1*, *PIK3CD*, *JUND*.

**Fig. 5:**
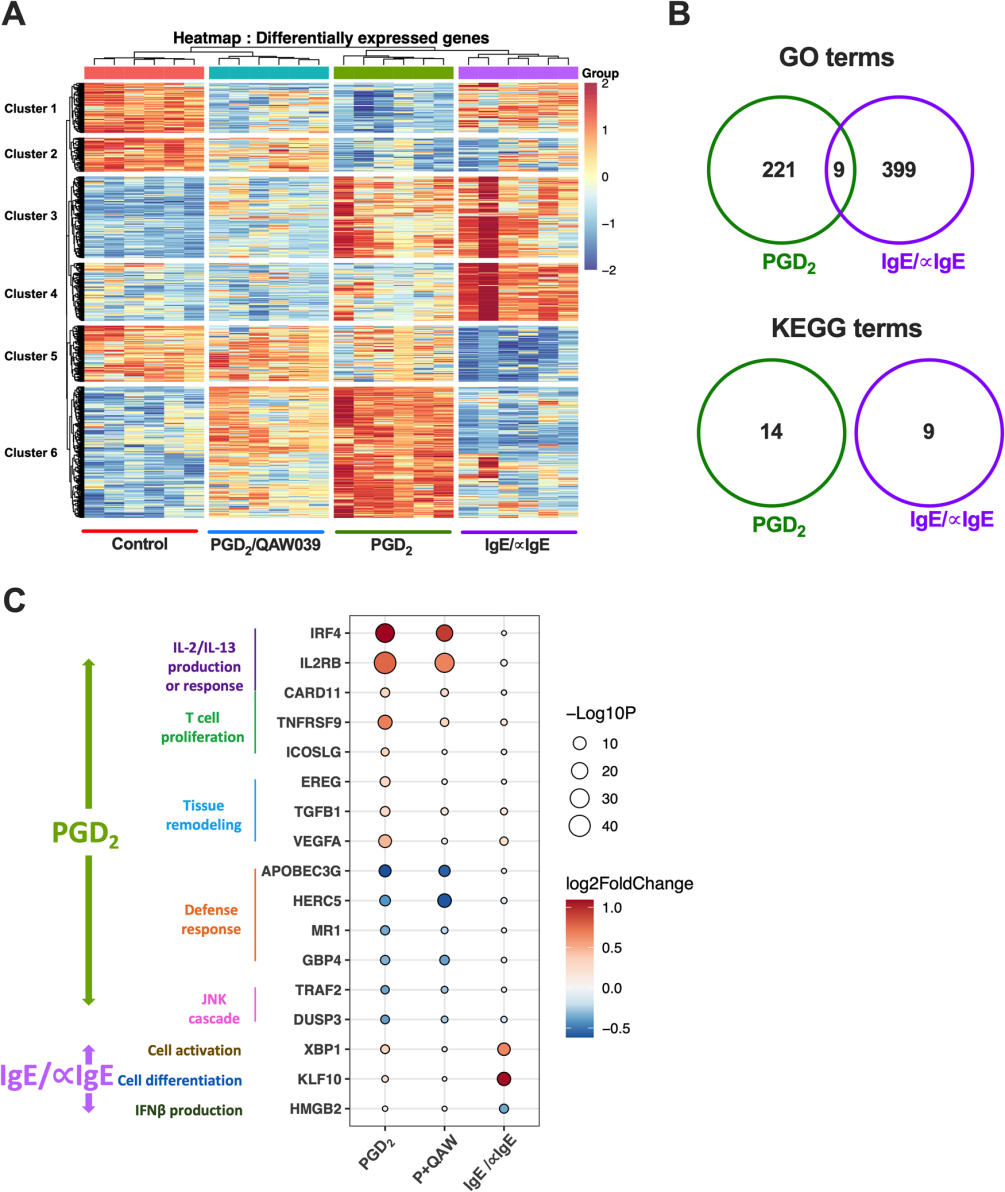
Distinct signatures and functions of PGD_2_ and IgE on basophils revealed by RNAseq. **A** Heatmap of clusters of differentially expressed genes among control, IgE/anti-IgE-, PGD_2_- and PGD_2_/QAW039-treated basophils. **B** Venn diagram indicating the numbers of GO- or KEGG-terms (*p* < 0.05) enriched differently by PGD_2_ or IgE/anti-IgE in basophils. **C** Key genes involved in different function modules from PGD_2_- and IgE/anti-IgE-treated basophils.

From the 1150 DEGs detected among all stimulation conditions (Control, PGD_2_, PGD_2_/QAW039, IgE/anti-IgE), 6 different clusters were found: Cluster 1 – DEGs downregulated only by PGD_2_; Cluster 2 – DEGs downregulated by both PGD_2_ and IgE/anti-IgE; Cluster 3 – DEGs upregulated by both PGD_2_ and IgE/anti-IgE; Cluster 4 – DEGs upregulated only by IgE/anti-IgE; Cluster 5 – DEGs downregulated only by IgE/anti-IgE; Cluster 6 – DEGs upregulated only by PGD_2_ (Fig. 5A). Within the DEGs regulated exclusively by PGD_2_ (Cluster 1 & 6) or IgE/anti-IgE (Cluster 4 & 5), gene set enrichment analyses identified 230 GO terms and 14 KEGG pathways for PGD_2_ stimulation, while IgE/anti-IgE stimulation was associated with 408 GO terms and 9 KEGG pathways, with only 9 GO terms shared by both (Fig. 5B). Semantic similarity analysis grouped these GO terms into six functional modules for PGD_2_ (Supplementary Fig. 8A) and IgE/anti-IgE (Supplementary Fig. 8B) stimulation, including upregulated cytokine production, T cell proliferation and tissue remodelling, as well as downregulated defence response for PGD_2_ stimulation, upregulated cell activation and differentiation and downregulated interferon gamma (IFN-β) production for IgE/anti-IgE stimulation. Key DEGs involved in each functional module are listed in Fig. 5C and Supplementary Fig. 8C.

### Effect of PGD_2_/DP2 in basophils is mediated by PI3K, ERK and p38 signalling pathways

**Fig. 6:**
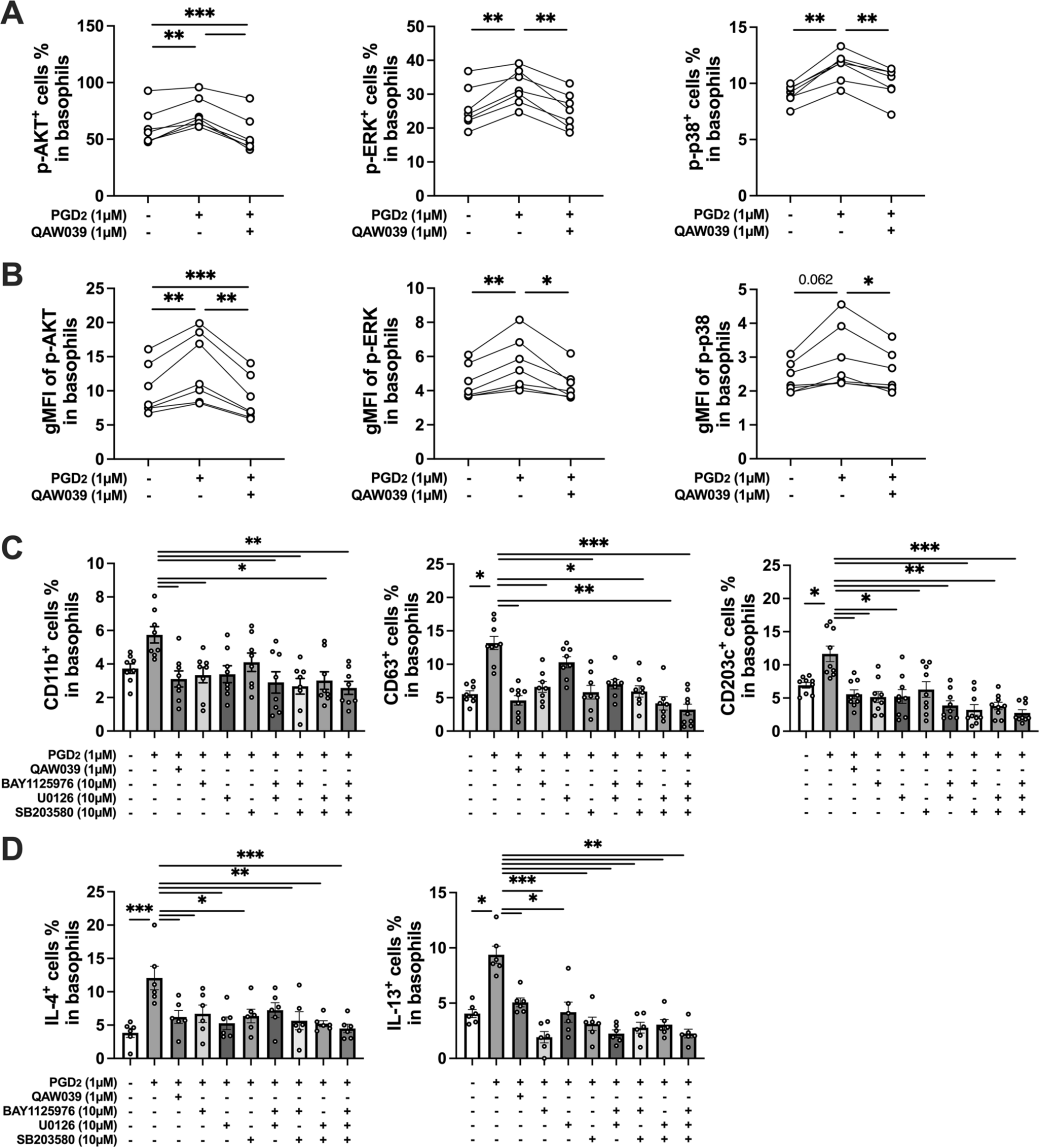
AKT, ERK and p38 signalling pathways in basophils were activated by PGD_2_ and reversed by DP2 antagonist. Frequencies of p-AKT, p-ERK and p-p38 positive cells in total basophils (**A**), and levels of these phosphoproteins in basophils (**B**) after treatment with 1 µM PGD_2_ in the presence of absence of 1 µM QAW039 for 1 h were measured with Phosflow in fresh blood. *n* = 7. Frequencies of CD11b, CD63 and CD203c positive basophils (**C**), and frequencies of IL-4- or IL-13-producing basophils (**D**) in fresh blood after incubation with PGD_2_ in the presence of various combinations of inhibitors of PI3K, ERK and p-38 signalling pathways (BAY1125976, U0126 or SB203580) were measured by flow cytometry (**C**) or intracellular cytokine staining (**D**). *n* = 8 for **C** and *n* = 6 for **D**. QAW039 was used as a control inhibitor. *p < 0.05, **p < 0.01, ***p < 0.001.

To validate the signal pathways detected by RNAseq in PGD_2_-activated basophils, we examined the phosphoproteins (p-AKT, p-ERK and p-p38) in basophils. The frequency of p-AKT^+^, p-ERK^+^ or p-p38^+^ basophils (Fig. 6A and Supplementary Fig. 9A) and the levels of these phosphoproteins (Fig. 6B) determined by Phospho-flow cytometry (PhosFlow) were increased by PGD_2_ stimulation and inhibited by QAW039.

To further explore the relative contributions of these signalling pathways to basophil activation, BAY1125976 (AKT inhibitor), U0126 (ERK inhibitor) and SB203580 (p38 inhibitor) were employed (Fig. 6C, D). These inhibitors partially reversed, and their combination almost completely blocked, the increase in activation markers CD11b, CD63 and CD203c (Fig. 6C) and IL-4/IL-13 production (Fig. 6D) induced by PGD_2_. The blockades seen were not due to drug-dependent cell death, as confirmed by survival analysis of cells post-treatments (Supplementary Fig. 9B). Therefore, these three pathways are required for PGD_2_/DP2-mediated basophil activation.

### PGD_2_/DP2 axis attenuates MR1-mediated antigen-presenting role of basophils

**Fig. 7:**
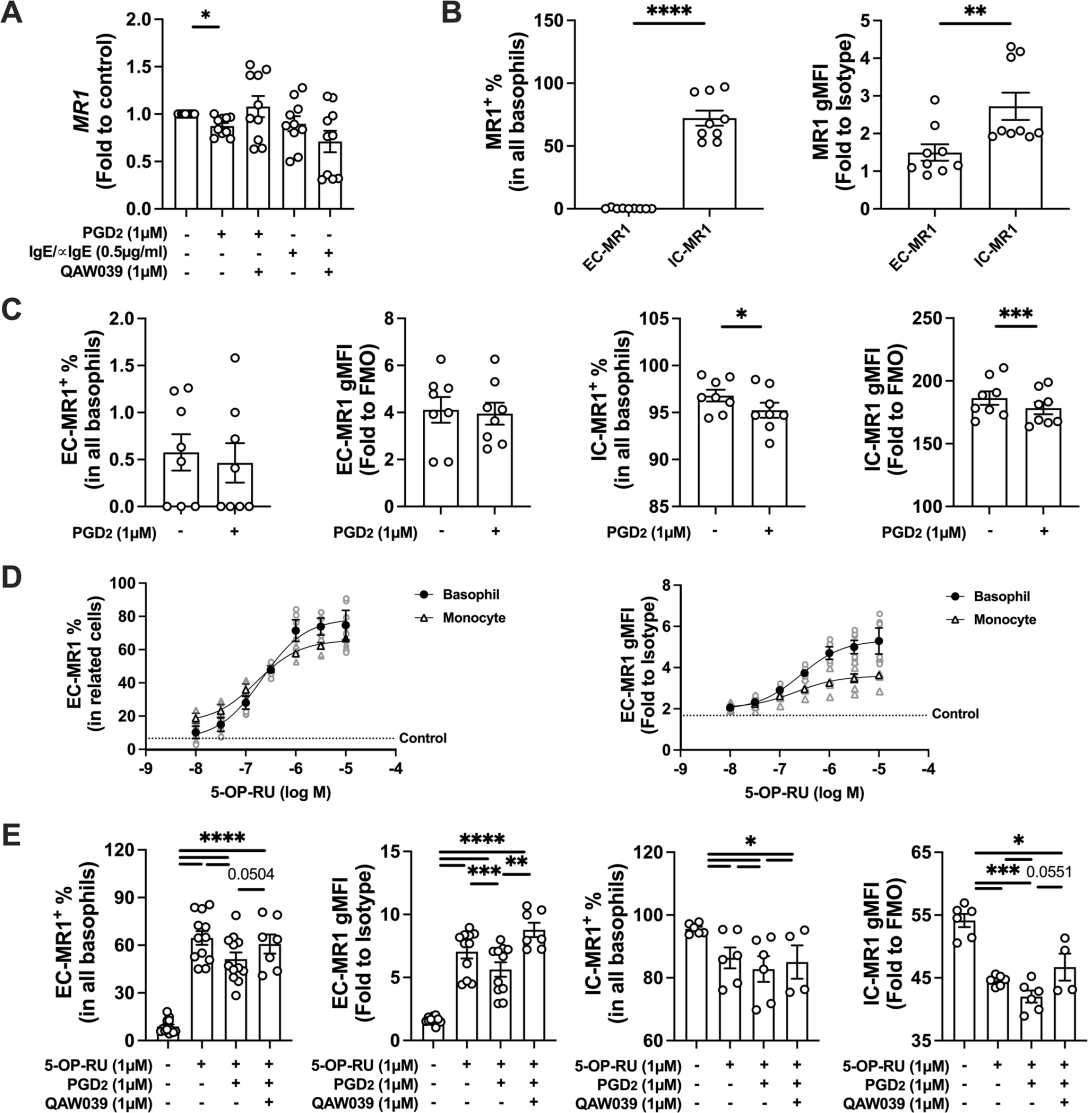
Upregulation of extracellular-MR1 on basophils by 5-OP-RU was reduced by PGD_2_. **A** Levels of MR1 mRNA in purified basophils after treatment with PGD_2_ or IgE in the absence or presence of QAW039 measured by qPCR. *n* = 10. **B** Membrane (EC-) and intracellular (IC-) MR1 in untreated basophils detected by flow cytometry. *n* = 9. **C** EC- and IC-MR1 expression in basophils after treatment with PGD_2_. *n* = 8. **D** Dose effect of 5-OP-RU on EC-MR1 expression on basophils and compared with that on monocytes from the same individuals. *n* = 6. **E** EC- and IC-MR1 expression in basophils in the presence of 5-OP-RU with or without pre-treatment of PGD_2_ and QAW039. *n* = 12 for control, 5-OP-RU or 5-OP-RU/PGD_2_, and *n* = 7 for 5-OP-RU/PGD_2_/QAW039 in left two panels; *n* = 6 for control, 5-OP-RU or 5-OP-RU/PGD_2_, and *n* = 4 for 5-OP-RU/PGD_2_/QAW039 in right two panels. *p < 0.05, **p < 0.01, ***p < 0.001, ****p < 0.0001.

Interestingly, pathway enrichment analysis of the RNAseq data identified downregulation of MR1, a non-classical antigen-presenting molecule that presents antigens to mucosal-associated invariant T cells (MAITs)^24^, by PGD_2_ but not IgE/anti-IgE (Fig. 5C and Supplementary Fig. 8C). To confirm the observation, we examined *MR1* mRNA levels in basophils via qPCR under the same treatment conditions as in the RNAseq experiment (Fig. 7A). The qPCR results were consistent with the RNAseq findings. We then investigated MR1 localization in basophils (Fig. 7B). Resting basophils exhibited no surface (extracellular, EC-) MR1, while MR1 predominantly localized intracellularly (IC-MR1), as expected. PGD_2_ had no effect on EC-MR1 levels but downregulated IC-MR1 at protein level (Fig. 7C). Previous studies suggest that MR1 accumulates in the endoplasmic reticulum (ER) in a ligand-receptive conformation and translocates to the cell surface upon antigen stimulation^25,26^. Treatment with the MR1 ligand 5-(2-oxopropylideneamino)-6-D-ribitylaminouracil (5-OP-RU) increased EC-MR1 on basophils with the dose of 5-OP-RU, peaking at ~1 µM after ~5 h (Fig. 7D and Supplementary Fig. 10A, B). This response was comparable to that on monocytes. IC-MR1 levels decreased after 5-OP-RU treatment (Fig. 7E), suggesting MR1 translocation. Pre-treatment with PGD_2_ partially reduced the 5-OP-RU-induced upregulation of EC-MR1 but had no further effect on IC-MR1 downregulation. The PGD_2_ effect was reversed by QAW039.

**Fig. 8:**
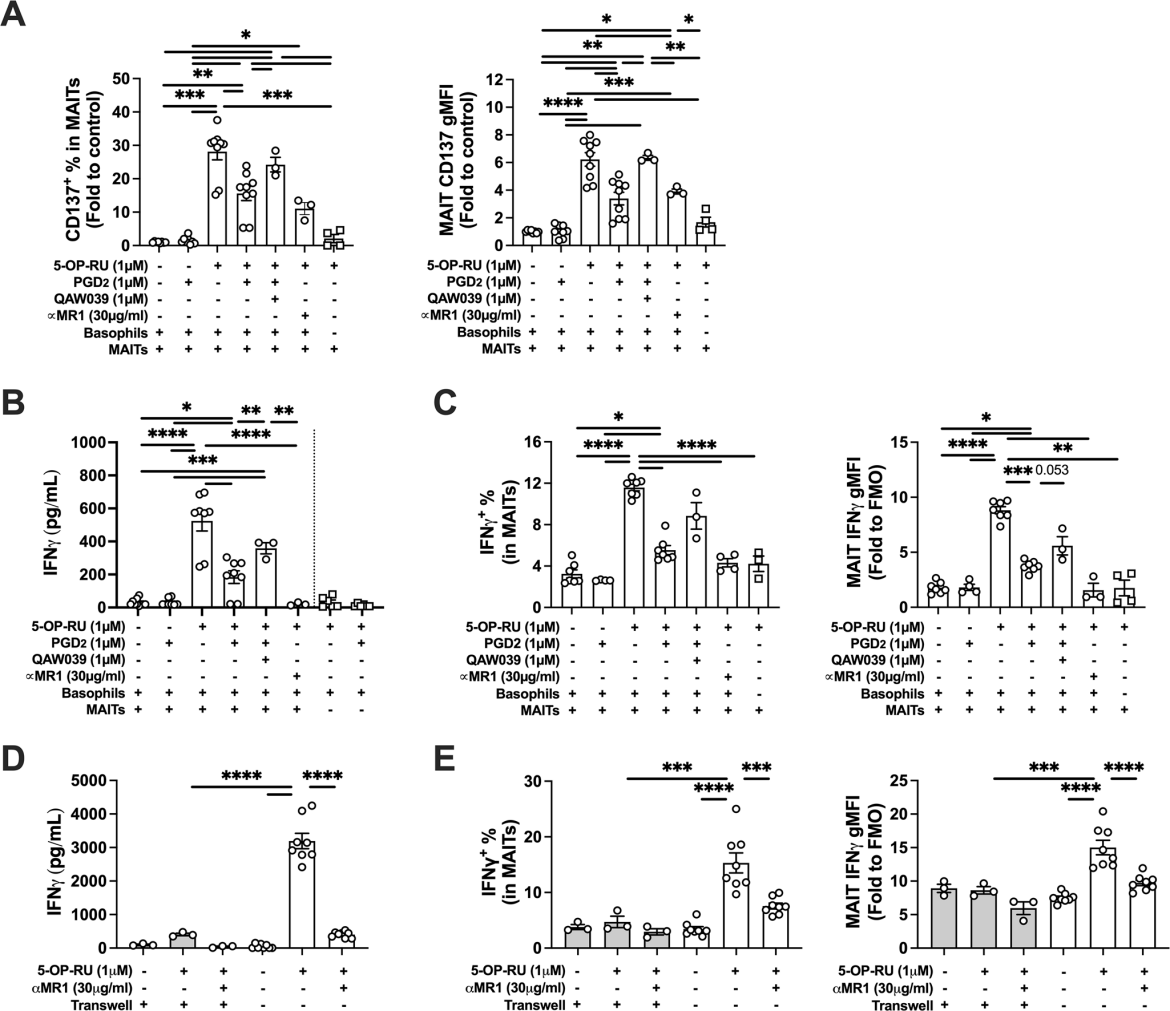
MR1-mediated antigen-presenting function of basophils in basophil/MAIT co-culture was impaired by PGD_2_. Basophils and MAIT cells, alone or co-cultured, in the presence or absence of 5-OP-RU, with or without PGD_2_ pre-treatment, with or without the addition of QAW039 in basophils. An anti-MR1 antibody was used as a control for MR1 blockade. **A** Expression of activation marker CD137 on MAIT cells was measured by flow cytometry. *n* = 9 for control, PGD_2_, 5-OP-RU or 5-OP-RU/PGD_2_; *n* = 3 for 5-OP-RU/PGD_2_/QAW039 or 5-OP-RU/∝MR1; *n* = 4 for 5-OP-RU/without basophil. **B** IFN-γ levels in the supernatants from the co-cultures or basophils alone were measured by ELISA. **C** IFN-γ^+^ MAIT cells and IFN-γ levels in gated MAIT cells from the co-cultures or MAIT cells alone were detected by flow cytometry. *n* = 8 for control, PGD_2_, 5-OP-RU or 5-OP-RU/PGD_2_; *n* = 3 for 5-OP-RU/PGD_2_/QAW039 or 5-OP-RU/∝MR1; *n* = 6 for 5-OP-RU/without MAITs or 5-OP-RU/PGD_2_/without MAITs. Basophils and MAIT cells were co-cultured, in the presence or absence of 5-OP-RU and anti-MR1 antibody, with or without a transwell insert to separate the two cell types. IFN-γ levels in supernatants were measured by ELISA (**D**), and IFN-γ^+^ MAIT cells and IFN-γ levels in gated MAIT cells were analysed by flow cytometry (**E**). *n* = 3 for all the conditions using transwell, and n = 8 for all the conditions without using transwell. *p < 0.05, **p < 0.01, ***p < 0.001, ****p < 0.0001.

To further investigate the effect of PGD_2_ on the MR1-mediated antigen-presenting function by basophils, we co-cultured basophils (with or without PGD_2_ pre-treatment, in the presence or absence of QAW039) with MAIT cells (Supplementary Fig. 11A) in the presence of 5-OP-RU and examined MAIT cell activation (Fig. 8). Co-culture of basophils with MAIT cells in the presence of 5-OP-RU increased the expression of CD137, a T cell activation marker associated with IFN-γ production by MAIT cells (Fig. 8A)^27^. Pre-treatment of basophils with PGD_2_ partially but significantly reduced the increase in CD137 on MAIT cells, an effect that was reversed by QAW039. Similar to CD137 expression in MAIT cells, IFN-γ production was induced by 5-OP-RU in the co-culture, and PGD_2_ significantly inhibited this production (Fig. 8B). The detected IFN-γ in the co-culture was contributed by MAIT cells, as basophils alone did not release IFN-γ and no IFN-γ^+^ basophil were detected in the co-culture (Fig. 8C and Supplementary Fig. 11B). The inhibitory effect of PGD_2_ on IFN-γ production by MAIT cells in the co-culture was mediated through DP2 as QAW039 reversed the effect of PGD_2_ (Fig. 8B, C), and mediated via MR1 as an anti-MR1 antibody blocked 5-OP-RU-induced MAIT cell activation (Fig. 8). Additionally, MAIT cells do not express DP2 (Supplementary Fig. 11C), and MAIT cells were not activated by 5-OP-RU in the absence of basophils (Fig. 8A, C). Similar results were also observed for TNFα, another cytokine produced by activated MAIT cells (Supplementary Fig. 11D).

To further substantiate the MR1-mediated antigen-presenting role of basophils to MAIT cells, co-culture experiments were conducted using transwell inserts to physically separate the two cell types (Fig. 8D, E and Supplementary Fig. 11E), as well as by exposing MAIT cells to basophil-conditioned medium without direct cell contact (Supplementary Fig. 11E–G). In both settings, MAIT cell activation (IFN-γ and TNFα production) was not detected, indicating that direct basophil and MAIT cell contact is required for MR1-dependent antigen-presentation.

To determine whether PGD_2_ exerts a direct inhibitory effect on MAIT cells, isolated MAIT cells were stimulated either in a TCR-independent manner (IL-12 and IL-18) or in a TCR-dependent manner (anti-CD3/CD28) in the presence of PGD_2_ (Supplementary Fig. 12). No detectable direct effect of PGD_2_ on MAIT cell activation markers (CD25, CD69 and CD137) was observed under either condition.

In addition, we examined basophil MR1 expression and MAIT cell profiles in patients with asthma (Supplementary Fig. 13). No significant difference in EC-MR1 expression on blood basophils was observed between healthy and asthmatic individuals (Supplementary Fig. 13A). Moreover, there was no correlation between basophil frequencies or counts and MAIT cell frequencies or counts in peripheral blood (Supplementary Fig. 13B).

## Discussion

Basophils are important players in allergic diseases, including asthma, via IgE/FcεR1 activation^2,28^. However, their paucity has made it challenging to fully understand their biological significance, particularly their IgE/FcεR1-independent role in asthma pathogenesis. Basophils express DP2 at high levels^6^. This study used the DP2 antagonist QAW039 to explore the PGD_2_/DP2-dependent pathogenic roles of basophils. We demonstrated that basophils are enriched in patients with severe eosinophilic asthma, independent of IgE levels, and is reversed by OCS. The PGD_2_/DP2 axis enables multiple proinflammatory functions in basophils, including promoting cell recruitment and activation, histamine and leukotriene production, and type 2 cytokine production, independent of IgE/FcεR1. RNAseq revealed that the effect of the PGD_2_/DP2 axis on basophils is mediated by PI3K/ERK/p38 signalling pathways, and that PGD_2_ and IgE induce distinct gene signatures. PGD_2_ specifically attenuates the antigen-presenting role of basophils by downregulating MR1, suggesting a previously unrecognized mechanism by which PGD_2_/DP2-dependent basophil activation could contribute to asthma pathogenesis.

Basophils are enriched in eosinophilic asthma and correlated with eosinophil counts^21^. Our data confirmed this association and further demonstrated that basophil counts increase with asthma severity. This enrichment of basophils was independent of age or gender in our cohort. IL-5 antagonism, which reduces disease severity and exacerbation in eosinophilic asthma, decreases not only eosinophil but also basophil levels. This suggests that basophils may contribute to the pathogenesis of severe eosinophilic asthma, although their role may be less prominent than that of eosinophils, as the reduction in basophil levels following mepolizumab treatment was less pronounced. The precise mechanism underlying the strong link between basophils and eosinophils remains unclear; however, these two cell types share several biological features. Both originate from the bone marrow, and express high levels of IL-5 receptor and CCR3, which are critical for cell growth, proliferation, activation and migration^29,30^. In humans, basophils appear to have a close lineage relationship with eosinophils, as cord blood-derived immature basophils exhibit a basophil–eosinophil hybrid phenotype^31^. Both cell types also respond to IL-3, IL-5, IL-33 during allergic inflammation. Another potential mechanism contributing to their correlation is the crosstalk between basophils and eosinophils, or with other immune cells such as Th2 cells. Cytokines and mediators released by activated basophils, such as TSLP, eotaxin, IL-4, IL-13 and cysLTs, may stimulate eosinophils or Th2 cells. Conversely, cytokines produced by Th2 cells, including IL-5, CSF1 and GM-CSF, may activate basophils or eosinophils. Further investigation in the future is required to confirm these hypotheses and elucidate the underlying mechanism.

Basophils could be activated by IgE-dependent or -independent stimuli. Crosslinking of IgE-bound-FcεR1 triggers strong basophil activation^32^. Omalizumab, an anti-IgE antibody used in allergic asthma treatment, affects basophil signalling, activation, and cytokine release^33^. It has been reported that basophils can be activated by IL-2, IL-3, GM-CSF and complement components (C3a/C5a) independent of IgE^34–36^. Our study broadly investigated the effect of PGD_2_ on basophils. Similar to its effect on other DP2^+^ cells^10,14,16,17^, PGD_2_ strongly activates human basophils, inducing migration, cytokine (IL-4/13) production, histamine and LTC_4_ release. These effects are mediated via DP2, as basophils lack DP1, and DP1 agonist or antagonist showed no effect on basophils, while DP2 antagonism completely blocks PGD_2_-induced responses. It has been suggested that, under allergic conditions, PGD_2_ is up-regulated at the early phase by activated mast cells, and that basophils are subsequently infiltrate to the affected tissue during the late phase, contributing to the second wave of histamine^37^. Our data support this hypothesis and provide further evidence demonstrating how basophils are recruited and activated by PGD_2_ during the late phase of diseases. RNAseq results suggested that PGD_2_ and IgE share similar effects in basophils in terms of cell activation, migration, promoting survival and response to metabolites and cytokines, but also have distinct functions. PGD_2_ stimulation is associated with impaired defence response to bacteria/viruses, tissue remodelling, and IL-2/IL-13 production, while IgE stimulation is associated with iron transport and IFN-β production. Although both PGD_2_ and IgE use similar signalling pathways, including PI3K and ERK, interestingly, PGD_2_, but not IgE, attenuates the antigen-presenting role of basophils to MAIT cells by downregulating MR1. Furthermore, our data suggested that basophil enrichment is not associated with IgE levels. Therefore, the PGD_2_/DP2 axis could contribute to the roles of basophils in asthma, particularly in IgE-low asthma.

The release of pro-inflammatory mediators is an important feature of activated basophils. Similar to mast cells, basophils release histamine upon IgE crosslinking or stimulation like IL-3^4^, evoking bronchoconstriction in asthma^38^. Although previous studies have reported that PGD₂ enhances, but does not independently induce, histamine release from human basophils^18,39^, we observed that PGD_2_ alone can trigger histamine release. This discrepancy may be attributable to differences in assay methods and/or samples (whole blood vs purified cells). Nevertheless, the effects of PGD_2_ on histamine release and CD63 upregulation are modest compared with its pronounced effects on basophil adhesion, migration, and cytokine production, indicating that PGD_2_ is not a strong inducer of basophil degranulation. Upon activation, mast cells produce both PGD_2_ and cysteinyl leukotrienes (cysLTs)^40^, whereas most studies suggest that basophils produce cysLTs but not PGD_2_^41^, although low levels of PGD_2_ production by basophils have been reported^42^. Our study confirmed that basophils generate cysLTs, particularly LTC_4_, albeit at lower levels than mast cells (~0.2 ng/10^6^ cell/ml vs. ~3.5 ng/10^6^ cell/ml)^43^. PGD_2_/DP2 axis enhanced LTC_4_ production in basophils, though its effect is weaker than IgE/anti-IgE stimulation. Neither PGD_2_ nor IgE/anti-IgE stimulation induced PGD_2_ production in our purified basophils. LTC_4_ is an arachidonic acid metabolite, plays an important role in the pathogenesis of allergic inflammations^3^. It is synthesized via the 5-lipoxygenase pathway of arachidonic acid metabolism, involving a cascade of enzymes including PLC, PLA2, 5-LO, FLAP and LTC4S. Alterations in the expression or activity of these enzymes may therefore regulate LTC_4_ production. Our results suggested that PGD_2_ and IgE/anti-IgE regulate LTC_4_ synthesis through partially distinct mechanisms, as PGD_2_, but not IgE/anti-IgE, upregulated LTC4S expression, whereas IgE/anti-IgE more strongly increased FLAP expression. It remains unclear whether post-translational regulation, such as phosphorylation or subcellular translocation of leukotriene-synthesizing enzymes, also contributes to the effects of PGD_2_ or IgE/anti-IgE. Further investigation is required to fully elucidate the mechanisms governing cysLT production in basophils.

MR1 is a non-classical antigen-presenting molecule highly conserved across most mammals. It presents small molecule antigens to a distinct subset of MR1-restricted T (MR1T) lymphocytes^24^. In humans, MR1 has been identified in multiple tissues, including the liver, lung and gut^44^, and in diverse cell types such as dendritic, B cells, and bronchial epithelial cells^25,45^. Under steady-state conditions, MR1 is primarily retained in the ER and traffics to the cell surface upon ligand exposure^25^. Our results showed a similar distribution in human basophils, where only IC-MR1 was readily detectable in resting basophils, but EC-MR1 was increased and IC-MR1 decreased after 5-OP-RU stimulation, suggesting a translocation of MR1 from the ER to the cell surface. PGD_2_ downregulated MR1 levels on 5-OP-RU-treated basophils, targeting the transcription level, as it also downregulated the mRNA of *MR1* and IC-MR1 in resting basophils. In contrast to the classical MHC-I pathway, which presents endogenous peptides to CD8^+^ T cells, the MR1-dependent pathway specifically presents small, non-peptide microbial metabolites to MAIT cells^24^. The antigen-presenting role of human basophils remains poorly defined. Our study reveals that human basophils express functional MR1 and can activate MAIT cells. Additionally, we found that MR1 expression can be downregulated in response to cellular activation.

MAIT cells, the dominant MR1T cell type in humans, constitute ~10% of human blood CD8^+^ T cells, recognize microbial antigen presented by MR1^+^ antigen-presenting cells and play an important role in antimicrobial and also antiviral immunity^46,47^. MAIT cells expand in the lungs of mice infected with pathogens, and produce IFN-γ, TNF, and IL-17A during the course of infection^46,48^. MAIT-deficient mice exhibit, for example, impaired T cell responses at the early stage of infection with Bacillus Calmette-Guérin^49^. In humans, a low frequency of blood MAIT cells in patients in the intensive-care unit correlated with the severity of acquired infections^50^, and reduced MAIT cell numbers in the blood and lung were associated with severe asthma^51^. However, the interactions between MAIT cells and basophils have never been previously reported. Here we demonstrated that basophils activate MAIT cells via an MR1/TCR mechanism. Although MAIT cells can also be activated in a TCR-independent manner by cytokines such as IL-12/IL-18 and IL-15^52^, we did not detect IL-12 or IL-15 production from basophils under our experimental conditions. Moreover, this activation was abolished by MR1-blocking antibodies or by physically separating basophils from MAIT cells, further confirming that MAIT cell activation by basophils is MR1-dependent. PGD_2_ reduced MAIT cell activation mediated by downregulating MR1 in basophils, while DP2 antagonism could reverse the effect of PGD_2_ and restore IFN-γ production by MAIT cells. We believe that the PGD_2_/DP2-mediated downregulation of MR1 in basophils is biologically interesting and functional, with the implication that, under certain disease conditions when PGD_2_ is produced, the PGD_2_/basophil axis could potentially impact resistance to microbial infection. In severe asthma, bacterial or viral infections are important causes of exacerbations^53^. Therefore, inhibition of PGD_2_/DP2 signalling could potentially maintain the MR1 level in basophils and promote the protective function of MAIT cells, supporting a reduction in the incidence of exacerbations in asthma. It has been observed that infections were more common in the placebo group than in the DP2 antagonist group in clinical trials^54^. Furthermore, clinical trials conducted by several pharmaceutical companies have shown a reduction in asthma exacerbations with DP2 antagonists^55–57^. Although this reduction of asthma exacerbations is commonly considered as a result of inhibition of eosinophil-mediated inflammation, our findings suggest that the PGD_2_/basophil/MR1/MAIT/IFN-γ axis might represent an unknown underlying mechanism that could contribute to this process. Besides basophils, other cell types that infiltrate the airways, such as macrophages and dendritic cells, can also express MR1^58^. Although it is difficult to directly compare the antigen-presenting capacities of these cells, previous studies have reported their comparable distributions in the submucosa of airways (basophils: 82 ± 14/mm^2^, macrophages: 150 ± 29/mm^2^, dendritic cells: 31.9 ± 11.98/mm^2^) from asthmatic patients^59–61^. Therefore, the antigen-presenting function of basophils discovered in this study is potentially of pathological significance. Additionally, it would be interesting to investigate whether other DP2^+^ cells, such as eosinophils and Th2 cells, have a similar regulatory pathway in future studies.

In summary, our study shows that basophils are enriched in severe asthma and are correlated with eosinophil counts. PGD_2_ activates basophils independently of IgE, promoting cell recruitment and pro-inflammatory mediator production. Although using similar signalling pathways, PGD_2_, unlike IgE, downregulates MR1 levels and attenuates a previously unrecognized antigen-presenting capacity of basophils. These findings reveal a previously unknown pathogenic mechanism of the PGD_2_/DP2/basophil axis contributing to asthma or other basophil-related diseases, particularly under IgE-low conditions.

## Methods

### Human clinical samples

Patients with severe eosinophilic asthma ( > 3% sputum eosinophils) and healthy controls were recruited from John Radcliffe Hospital, Oxford (Supplementary Table 1)^62^. OCS users were defined as those who had taken any doses of OCS within seven days before recruitment. Peripheral blood was collected in EDTA tubes. Sputum was induced with nebulized saline (3–5%, Laboratoire UNITHER) after salbutamol (IVAX Pharmaceuticals Ireland) pre-treatment. Sputum plugs were dispersed with 0.2% dithiothreitol (D9779, Sigma-Aldrich), filtered to obtain cells for flow cytometry.

Ethical approval was granted by the South Central-Oxford B Research Ethics Committee, UK (18/SC/0361). All relevant ethical regulations were followed, and written informed consent was obtained from all participants.

### Human basophil preparation and treatment

Basophil-enriched fractions were obtained from human leucocyte cone (NHS Blood and Transplant, Oxford, UK) using a Percoll (17-0891-01, VWR International Ltd) gradient with two densities (1.07 g/ml and 1.08 g/ml). Basophils were then isolated with a MACS Human Basophil Isolation Kit (130-092-662, Meltenyi Biotec). The purity of the cells was checked by flow cytometry using antibody Panel 1 (Table S2) and cytospin staining with Rapid Diff II staining kit (RRSP134-D/135-D, Atom Scientific). Purified basophils (>95% purity) were resuspended in RPMI 1640 (21875-034, Gibco) with 10% human serum (H4522, Sigma-Aldrich) and 10 IU/ml IL-3 (130-093-908, Miltenyi Biotec) and used immediately for assays or basophil/MAIT co-culture.

For cytokine/protein assays, basophils were incubated with 1 µM PGD_2_ (12010, Cayman Chemical) in the presence or absence of various concentrations of QAW039 (Novartis Pharma AG), 1 µM BW245C (12050, Cayman Chemical), 1 µM BW868C (12060, Cayman Chemical), or 0.5 µg/ml human IgE (AG30P, Chemicon International)/0.5 µg/ml anti-IgE antibody (I6284, Sigma-Aldrich) for 4 h. To differentiate the stimulator from the product PGD_2_, DK-PGD_2_ (12610, Cayman Chemical), a specific DP2 agonist undetectable in the PGD_2_ ELISA, was used to stimulate cells for PGD_2_ assay ^14^. Supernatants were harvested for ELISA, and pellets were stored at −80 °C for qPCR and RNAseq.

### Flow cytometry

For basophil profile, fresh blood was labelled with antibody Panel 2 (Supplementary Table 2), followed by red blood cell lysis and fixation with a BD FACS Lysing Solution (349202, BD). Sputum cells were stained similarly but included live/dead marker Zombie Aqua (423102, BioLegend).

For ex vivo intracellular cytokine staining, PBMCs isolated from fresh blood were incubated with 1 µM PGD_2_ or 25 ng/ml PMA (P8139, Sigma-Aldrich)/1 µg/ml ionomycin (I0634, Sigma-Aldrich) in the absence or presence of 1 µM QAW039, 10 µM BAY1125976 (28913, Cayman Chemical), 10 µM U0126 (662005, Calbiochem) or 10 µM SB203580 (S8307, Calbiochem) for 6 h. Brefeldin A (5 µg/ml, 420601, BioLegend) was added at 30 min after the start of stimulation. Cells were stained with an antibody Panel 3 (Supplementary Table 2), followed by fixation with IC fixation buffer (00-8222-49, Invitrogen) overnight at 4 °C. The cells were treated with permeabilization buffer (00-8333-56, eBioscience) followed by intracellular staining with antibody Panel 4 (Supplementary Table 2), and analysed for CD123^+^HLA-DR^-^IL-4/13^+^ cells.

For basophil activation marker analysis, fresh blood was treated with PGD_2_ in the absence or presence of 1 µM QAW039, 10 µM BAY1125976, 10 µM U0126 or 10 µM SB203580 for 20 min, washed with cold PBS (14190-094, Gibco), stained with antibody Panel 5 (Supplementary Table 2), followed by red blood cell lysis and fixation with a BD FACS Lysing Solution.

For Phospho-flow cytometry (Phosflow), phosphoproteins (p-AKT, p-ERK1/2 and p-p38) in basophils were measured following BD Phosflow^TM^ protocol. Cell pellets from fixed whole blood samples (stimulated with or without 1 µM of PGD_2_ ± 1 µM QAW039 for 2 h) were permeabilized with Perm Buffer II (558052, BD Biosciences), and then stained with antibody Panel 6 (Supplementary Table 2).

For MR1 staining, PBMCs were incubated with 5-OP-RU (gift from Jeffery Mak) for 5 h for the dose curve or pre-treated with 1 µM PGD_2_ for 4 h before 1 µM 5-OP-RU for an additional 5 h. The cells were washed with PBS and stained with antibody Panel 7 (Supplementary Table 2). For intracellular MR1, cells were further fixed and permeabilized using FOXP3 Fix/Perm Buffer Set (421403, BioLegend) and stained with MR1 antibody conjugated with a different fluorochrome.

Data were acquired by BD FACSDiva (v8.0.1) on BD LSRFortessa or LSRII, or SpectroFlo (v3.3.0) on Cytek Aurora flow cytometers.

### Co-culture of basophils and MAIT cells

MAIT cell lines were prepared by FACS-sorting MAIT cells from human PBMCs using CD3 and MR1 Tetramer staining, expanded using phytohemagglutinin (10082333, Thermo Scientific), IL-2 (200-02, PeproTech) and γ-irradiated PBMC feeders, then maintained in RPMI 1640 containing AB human serum, IL-2, Penicillin/Streptomycin (P0781, Sigma-Aldrich), sodium pyruvate (11360070, Gibco), non-essential amino acids (12084947, Gibco)^63^. Before co-culture, basophils were treated with or without 1 µM PGD_2_ for 4 h, followed by the addition of 1 µM 5-OP-RU and MAIT cells at a basophils:MAITs ratio = 5:1 and incubation for 18 h. For transwell experiments, basophils were treated with 1 µM 5-OP-RU and seeded in the bottom chamber of a Millicell^®^ 96-well plate (pore size 0.4 µm, PSHT004R1, Millipore), while MAIT cells were loaded on the upper chamber. MR1 antibody (32549, Cayman Chemical) was used to block the function of MR1 in some controls. Supernatants were stored at -80 °C for ELISA. Cells were stained with antibody Panel 8 (Supplementary Table 2) to determine activation markers on MAIT cells, or antibody Panel 9 (Supplementary Table 2) for intracellular staining of IFN-γ.

### Chemotaxis

Isolated basophils were resuspended in RPMI 1640 containing 10% human serum and then incubated with QAW039, 1 µM BW245C, 1 µM BW868C or medium alone in the upper chamber (5 µm pore) of HTS Transwell 96-well permeable supports (3388, Corning) for 1 h at 37°C. The upper chamber was then placed into the lower chamber containing medium, 100 nM PGD_2_ or 1 µM BW245C for another hour. Cell migration to the lower chamber was quantified using IncuCyte ZOOM.

### ELISA

IL-4 and IL-13 levels in basophil supernatants, as well as IFN-γ, IL-12 and IL-15 levels in the supernatants of basophils/MAIT cell co-cultures, were measured using ELISA kits (88-7046-86 for IL-4 and 88-7439-88 for IL-13, Invitrogen) or ELISA MAX kits (430104 for IFN-γ, 431704 for IL-12 and 435104 for IL-15, BioLegend). Histamine, LTC_4_ and PGD_2_ concentrations in the supernatants were determined with a competitive Human Histamine ELISA kit (ab213975, Abcam), an LTC_4_ enzyme immunoassay kit (501070, Cayman Chemical), or a PGD_2_-MOX enzyme immunoassay kit (512011, Cayman Chemical), respectively. The results were recorded using SPECTROstar (v2.10) on EnVision Multilable Reader (PerkinElmer), or Galapagos (v1.3.0.0) on EZ Read 400 Microplate Reader (Biochrom).

### qPCR

Total RNA from basophil pellets was extracted with RNeasy Plus Mini Kit (74134, Qiagen) and converted to cDNA with High-Capacity cDNA Reverse Transcription Kit (4374966, Applied Biosystems). qPCR was performed using Fast SYBR Green Master Mix (4385617, ThermoFisher) in a CFX96^TM^ Real-Time System (Bio-Rad CFX Manager v3.1). The oligo (primer) sequences used in qPCR are listed in Supplementary Table 3. *GAPDH* served as the reference gene.

### RNAseq

RNA from basophils (*n* = 6) was extracted using the RNeasy Plus Mini kit, and RNAseq was performed on a NovaSeq platform (Novaseq6000; paired-end 150 bp reads, ~10 million reads/sample) by Novogene Company Limited, Cambridge, UK. Data were analysed following *DSeq2* pipeline in R (v4.2.2)^64^. Data were analysed following *DSeq2* pipeline in R (v4.2.2). Pre-filtering was performed by retaining genes with ≥40 counts in ≥90% of the samples within the group with the smallest gene counts. Batch effects were adjusted using *ComBat-seq* with negative binomial regression to model batch effects across different biological samples^65^. Clustering was visualized using PCA for dimensionality reduction on the normalized data with variance-stabilizing transformations. DEGs among stimulation groups and between individual pair-wise comparisons were identified using the likelihood ratio test (LRT) and Wald test, respectively, with log-fold change shrinkage via the *apeglm* method and multiple testing correction. DEGs were further analysed for biological functions and pathway profiles using *clusterProfiler* in GO and KEGG databases. Functionally similar biological functions and pathways were clustered using GO semantic similarity analysis. Raw data are available at the Gene Expression Omnibus (Accession ID: GSE328453).

### Cell aggregation

Cell aggregation was imaged using a Nikon Eclipse TS100 microscope and analysed using Trainable Weka Segmentation. Cell clumps were identified via Otsu thresholding method. The intensity and size were measured. Results were reported as integrated intensity.

### Statistics and reproducibility

Reproducibility of the study findings was confirmed across at least three independent experiments using independent biological samples. Continuous data were analysed using one-way ANOVA with Tukey’s post hoc test or student’s *t*-tests, while dichotomous data were analysed using the chi-square test. *p*-values < 0.05 were considered statistically significant. Correlation coefficients (*R*_*s*_) were calculated using Spearman’s rank correlation test. Flow cytometry data were analysed using FlowJo software (v10.8.1). All statistical analyses were performed using GraphPad Prism (v10.3.1) or R (v4.2.2). Data are presented as mean ± SEM. Each data point in the figures represents a biologically independent sample.

### Reporting summary

Further information on research design is available in the Nature Portfolio Reporting Summary linked to this article.

## Supplementary information

Supplementary Information

Reporting summary

Transparent Peer Review file

## Data Availability

The bulk-RNAseq datasets generated and/or analysed during the current study are available in the Gene Expression Omnibus (Accession ID: GSE328453). The source data are available in the Oxford Research Archive repository (10.5287/ora-kzy490n9w). All other data are available from the corresponding authors upon reasonable request.
