# Supplementary material for: The PGD2/DP2 axis promotes pro-inflammatory responses but attenuates an antigen-presenting role of human basophils
Source: Commun Biol. 2026 May 20;9:1079. doi: 10.1038/s42003-026-10286-w (PMC13457600; doi:10.1038/s42003-026-10286-w)
Supplement: Supplementary file 1 [file 42003_2026_10286_MOESM1_ESM.pdf]

SUPPLEMENTARY INFORMATION

The PGD_2/DP2 axis promotes pro-inflammatory responses but attenuates an antigen-presenting role of human basophils

Luo J, Chen W, Lett MJ, Yu S, Luo W, Coakley E, Kazani S, Shikatani EA, Hinks TSC, Sandham DA, Pavord ID, Klenerman P, and Xue L.

SUPPLEMENTARY METHODS

Treatment for Flow cytometry

For MAIT cell quantification, PBMCs were labelled with antibody Panel 10 (Supplementary Table 2) and fixed using IC fixation buffer (Invitrogen).

For PGD_2/MAIT cell interaction assay, MAIT cells were either stimulated with 50 ng/ml IL-12 (219-IL, R&D Systems) and IL-18 (9124-IL, R&D Systems) in the absence or presence of 1 µM PGD_2, or incubated in anti-CD3 (2 µg/ml, OKT3, 317326, BioLegend) and anti-CD28 (2 µg/ml, CD28.2, 302934, BioLegend)-coated 96-well plates with or without 1 µM PGD_2 for 18 h at 37 °C, and then followed by staining with antibody Panel 11 (Supplementary Table 2).

ELISA for LTE_4 and TNFα

LTE_4 and TNFα levels were measured by using LTE_4 enzyme immunoassay kit (501060, Cayman Chemical) or TNFα ELISA MAX kits (430204, BioLegend), respectively, following the manufacturer's instruction.

Treatment of MAIT cells with basophil conditioned medium

Purified basophils were stimulated with 1 µM 5-OP-RU in the presence or absence of 30 µg/ml anti-MR1 antibody for 5 h. The conditioned medium was collected by harvesting the supernatant after centrifugation to remove cells. MAIT cells were then incubated with conditioned medium for 18 h. Supernatants were analysed by ELISA for IFN-γ and TNF-α. Cells were stained with antibody Panel 9 (Supplementary Table 2) for intracellular staining of IFN-γ.

SUPPLEMENTARY TABLES

Supplementary Table 1. Study subjects (Mean ± SD)

Characteristic	Control	Asthma		*p*-value	Asthma
		Non-eosinophilic	Eosinophilic		for Fig. 1F-G
	(n=28)	(n=25)	(n=51)		(n=60)
Age (y)	39.1±11.7	54.8±13.2	56.1±14.3	<0.001	55.9±15.1
Sex (male%)	29	59	51	0.054	41
Atopy (%)	14	46	54	0.002	55
BMI	24±5.6	28±5.5	30±6.5	0.072	30±8.3
FEV1 (% pred)	80.1±5.6	64.2±16.2	63.5±13.7	<0.001	69.6±42.3
Sputum eosinophils (%)	0.36±0.45	0.95±0.73	27.57±26.2	<0.0001	24.69±31.46
Blood eosinophils (10^9/L)	0.11±0.06	0.14±0.08	0.50±0.36	<0.0001	0.40±0.32
FeNO (ppb)	25±21	26±15	49±40	<0.05	51±46
Serum IgE (IU/mL)	23.8±29.5	225.6±328.0	470.7±693.3	<0.05	566.0±1057
ICS dose (µg)	0	1228±621	1548±597	<0.0001	1779±606
OCS (yes/no)	0/28	4/21	9/42		24/35
Mepolizumab (yes/no)	0/28	0/25	0/51		60/0
Dupilumab (yes/no)	0/28	0/25	0/51		0/60

BMI, body mass index; FeNO, fractional exhaled nitric oxide; FEV1, forced expiratory volume in one second; ICS, inhaled corticosteroids; OCS, oral corticosteroids; SD, standard deviation.

Supplementary Table 2. Antibodies used for flow cytometry

Antigen	Clone	Catalogue No.	Supplier	Antibody panels*
CD3	OKT3	317306	BioLegend	1,8,9
CD3	SK7	130-129-580	Miltenyi Biotec	11
CD11b	ICRF44	12-0118-41	eBioscience	5
CD11b	CBRM1/5	301403	BioLegend	5
CD14	TuK4	MHCD1417	Life technologies	1
CD19	SJ25C1	25-0198-42	eBioscience	1
CD25	BC96	302606	BioLegend	11
CD41	HIP8	303703	BioLegend	1
CD63	H5C6	353008	BioLegend	5
CD69	FN50	310906	BioLegend	8
CD69	FN50	130-112-615	Miltenyi Biotec	11
CD123	32703	FAB301C	R&D	2,3,5,6,7,8,9
CD137	4B4-1	309804	BioLegend	8
CD137	4B4-1	309809	BioLegend	11
CD161	191B8	130-113-598	Miltenyi Biotec	10
CD161	191B8	130-113-594	Miltenyi Biotec	11
CD193	5E8	310708	BioLegend	1,2,8,9
CD203c	NP4D6	324612	BioLegend	5
DP2	BM16	130-113-600	Miltenyi Biotec	1,2,5
FcεRI	AER-37 (CRA-1)	334616	BioLegend	1,2
HLA-DR	Tu36	361612	BioLegend	3,5,6,7
IFN-γ	B27	506524	BioLegend	9
IL-4	MP4-25D2	500826	BioLegend	4
IL-5	JES1-39D10	500904	BioLegend	4
IL-13	85BRD	11-7136-42	Invitrogen	4
p-AKT	M89-61	560343	BD	6
p-ERK	20A	612593	BD	6
p-p38	36/p38	612595	BD	6
MR1	26.5	361107	BioLegend	7
MR1	26.5	361105	BioLegend	7
TCR Vα7.2	3C10	351714	BioLegend	10
TCR Vα7.2	3C10	351703	BioLegend	11
Zombie Aqua		423102	BioLegend	1,2,3,5,6,7,8,9,10

*Panel 1 for basophil purity check;
*Panel 2 for basophil profile;
*Panel 3 for basophil identification before ICS;
*Panel 4 for IL-4/13 ICS;
*Panel 5 for basophil activation marker staining;
*Panel 6 for Phosflow staining;
*Panel 7 for MR1 staining;
*Panel 8 for MAIT activation marker staining;
*Panel 9 for IFN-γ ICS;
*Panel 10 for MAIT cell staining;
*Panel 11 for PGD_2/MAIT cell interaction assay.

Supplementary Table 3. Primers used for quantitative PCR

Gene	Primer *
ALOX5	F:5’-CTCAAGCAAGCAACACCGACGTAAA-3’<br>R:5’-CCTTGTGGCATTTGGCATCG-3’
ALOX5AP	F:5’-TTGCCTTTGAGCGGGTCTAC-3’<br>R:5’-GCCTCACAAACAAGTACATCAGT-3’
GAPDH	F:5’-AGCCACATCGCTCAGACAC-3’<br>R:5’-GCCCAATACGACCAAATCC-3’
IL4	F:5’-CACCGAGTTGACCGTAACAG-3’<br>R:5’-GCCCTGCAGAAGGTTTCC-3’
IL13	F:5’-AGCCCTCAGGGAGCTCAT-3’<br>R:5’-CTCCATACCATGCTGCCATT-3’
MR1	F:5’-GATTGAGCAAGCTGAGTGGC-3’<br>R:5’-CACACAAGGCCTTCTCCCAA-3’
PLA2	F:5’-CCTCCCCACCCTGAAAAA-3’<br>R:5’-TGCCAAAATTTTAAATGGAACAC-3’
PLC	F:5’-CAGTGTTAATGAGAACCACTCCA-3’<br>R:5’-TTGCTGACTCTCTTCTCTCTTAACC-3’
PTGDR1	F:5’-CCTGGAGGAGCTGGATCA-3’<br>R:5’-GCTCCATAGTAAGCGCGATAAA-3’
PTGDR2	F:5’-CCTGTGCTCCCTCTGTGC-3’<br>R:5’-TCTGGAGACGGCTCATCTG-3’

*All the primers were designed with Roche Universal ProbeLibrary or NIH Primer-BLAST and synthesized by ThermoFisher Scientific.

SUPPLEMENTARY FIGURES

Suppl. Figure 1

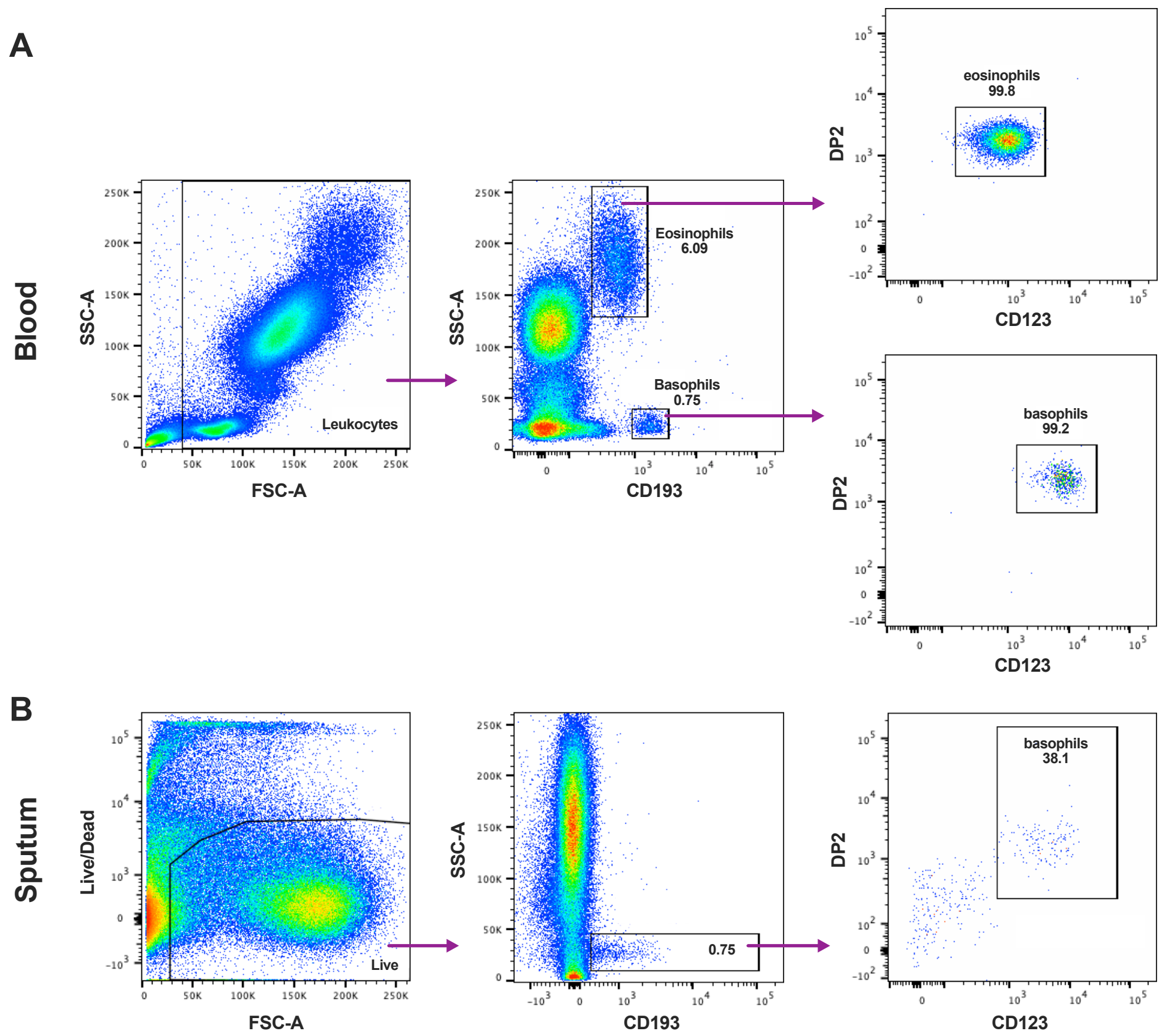

Supplementary Fig. 1 Gating strategies for basophils in peripheral blood or in induced sputum. Fresh bloods (**A**) or cell pellets from sputa (**B**) were stained with a mixture of antibodies and analysed with flow cytometry. Basophils were gated as $SSC^{low}CD193^{+}CD123^{+}DP2^{high}$ cells. Eosinophils were gated as $SSC^{high}CD193^{+}CD123^{+}DP2^{high}$ cells.

Suppl. Figure 2

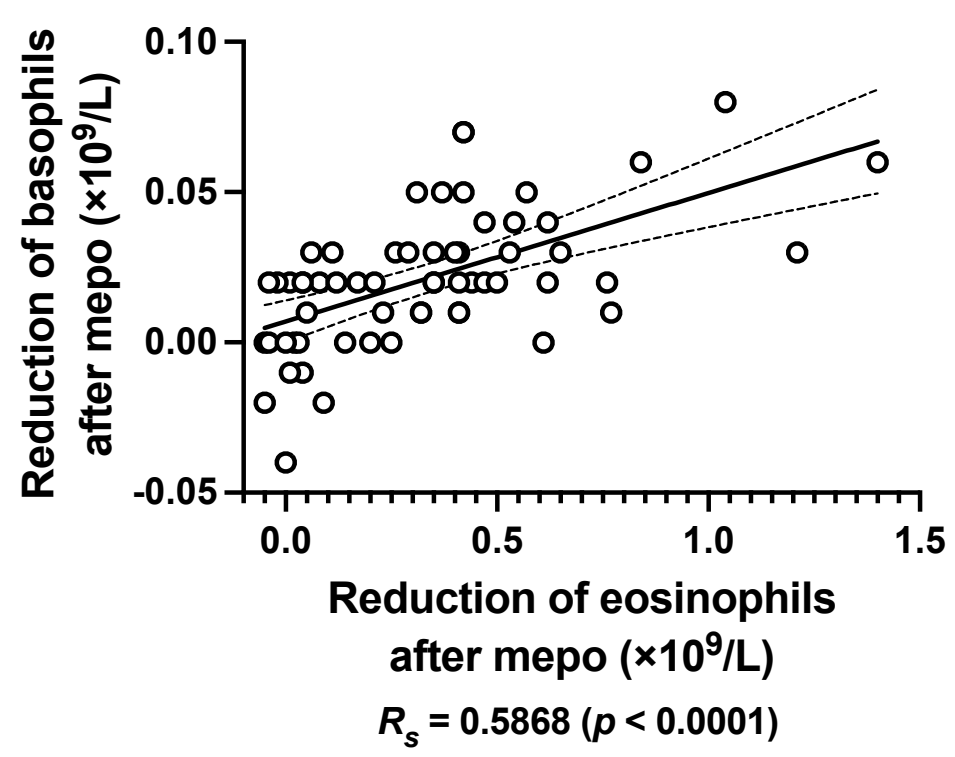

Supplementary Fig. 2 Correlation analysis of eosinophil downregulation and basophil downregulation after mepolizumab treatment. ****p<0.0001. n=60.

Suppl. Figure 3

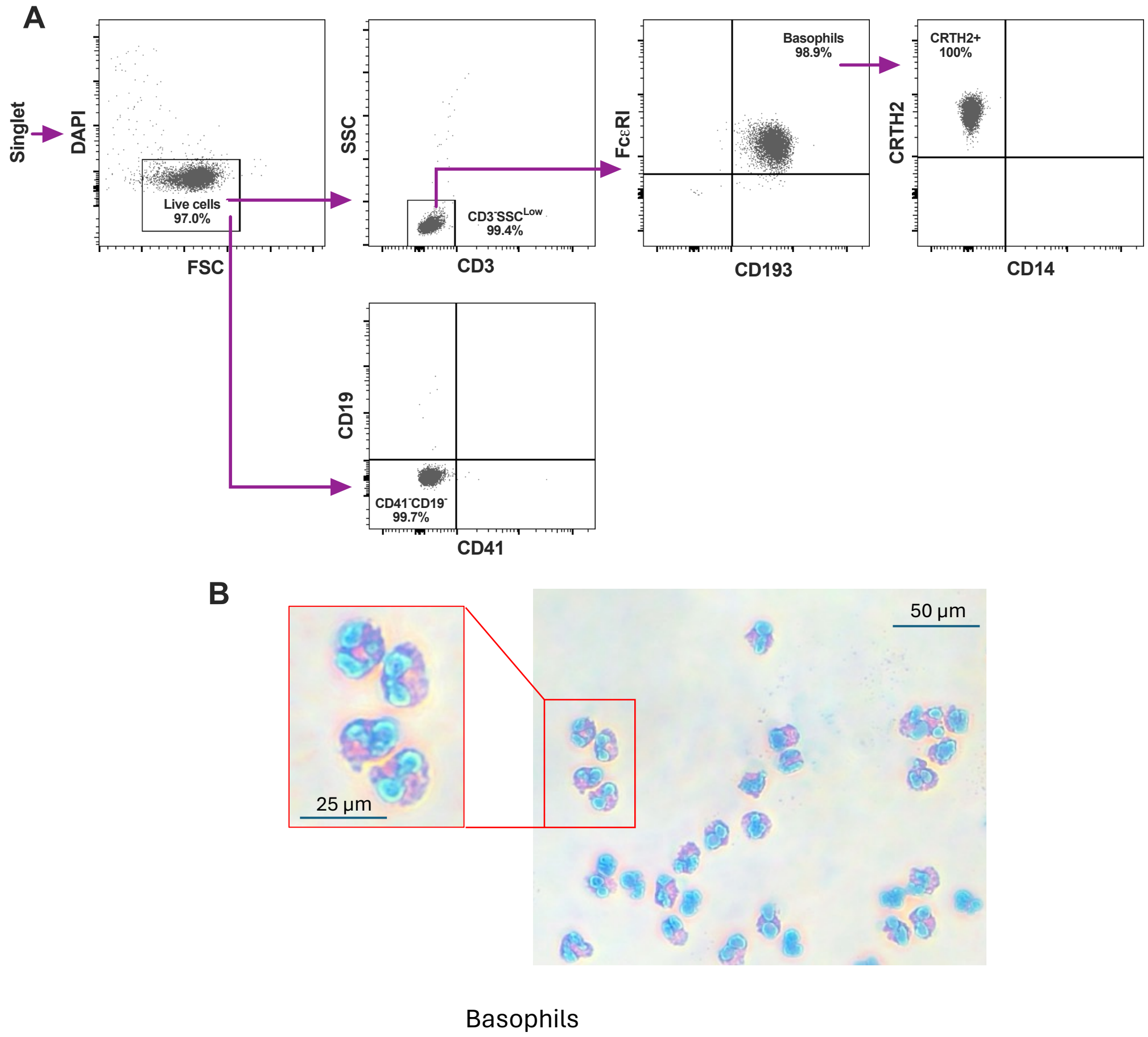

Supplementary Fig. 3 Purity of isolated basophils. **A** The purity of isolated basophils was tested with flow cytometry. **B** Isolated basophils were stained with Rapi-Diff II staining kit.

Suppl. Figure 4

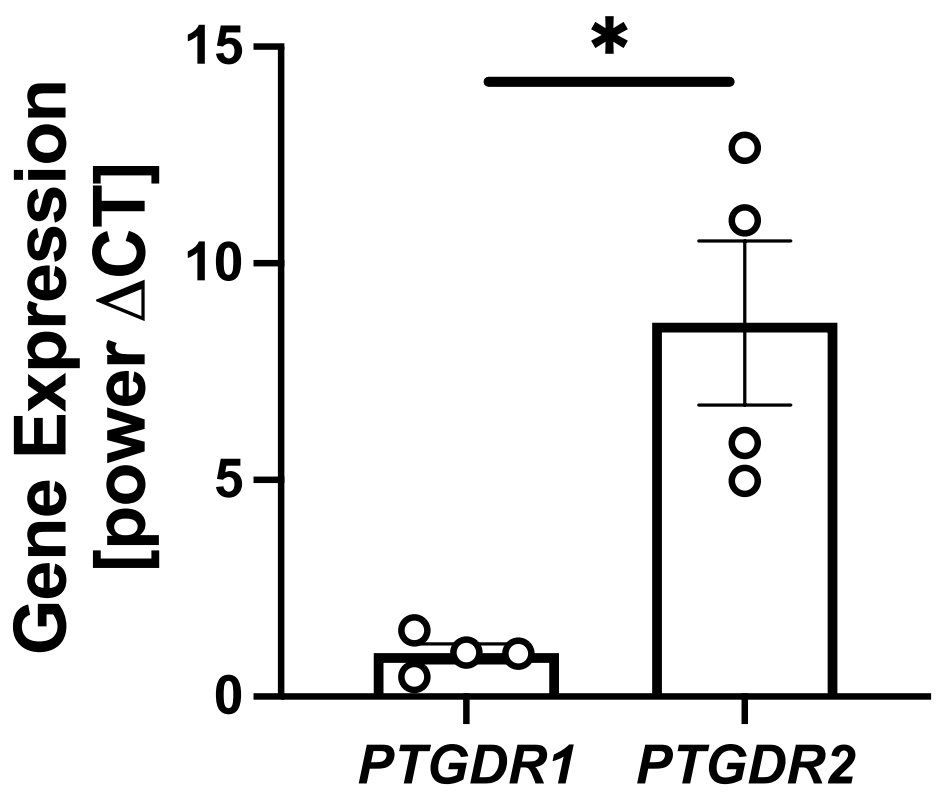

Supplementary Fig. 4 The levels of mRNA for the PGD_2 receptors (*PTGDR1* and *PTGDR2*) in basophils were compared by using qPCR. *p<0.05. n=4.

Suppl. Figure 5

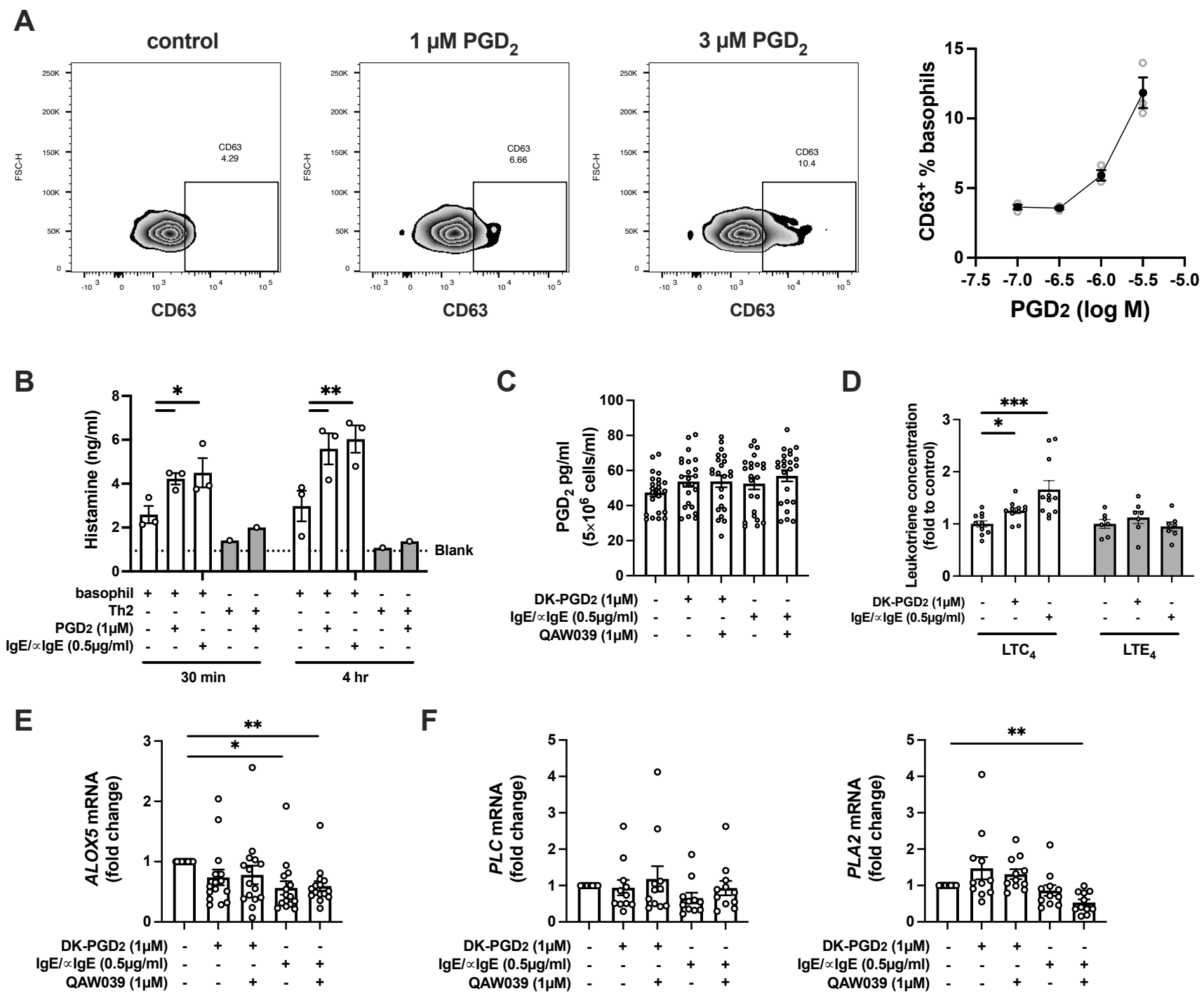

Supplementary Fig. 5 Effect of PGD_2/DP2 and IgE on degranulation and prostanoid synthesis in basophils. **A** Level of CD63 on the surface of basophils gated from whole blood was increased after PGD_2-treatment in a dose-dependent manner detected by flow cytometry. n=4. **B** Levels of histamine in the supernatants of isolated basophils after treated with PGD_2 or IgE/anti-IgE for 0.5 or 4 h measured with ELISA. Th2 cells were used as negative control. n=3 for conditions using basophils, and n=1 for Th2 controls. **C** Levels of PGD_2 in the supernatants of isolated basophils after treatments with DK-PGD_2 or IgE/anti-IgE in the presence or absence of QAW039 detected using ELISA. n=23. **D** Levels of LTC_4 and LTE_4 in the supernatants of basophils after treatments with DK-PGD_2 or IgE/anti-IgE were compared with ELISA. n=11 for LTC_4, and n=7 for LTE_4. **E** and **F** mRNA levels of *ALOX5* (gene for 5-lipoxygenase) (**E**), *PLC* (gene for phospholipase C) and *PLA2* (gene for phospholipase A2) (**F**) in basophils after treatments with DK-PGD_2 or IgE/anti-IgE in the presence or absence of QAW039 determined with qPCR. n=15 for **E**, and n=11 for **F**. **p*<0.05, ***p*<0.01, ****p*<0.001.

Suppl. Figure 6

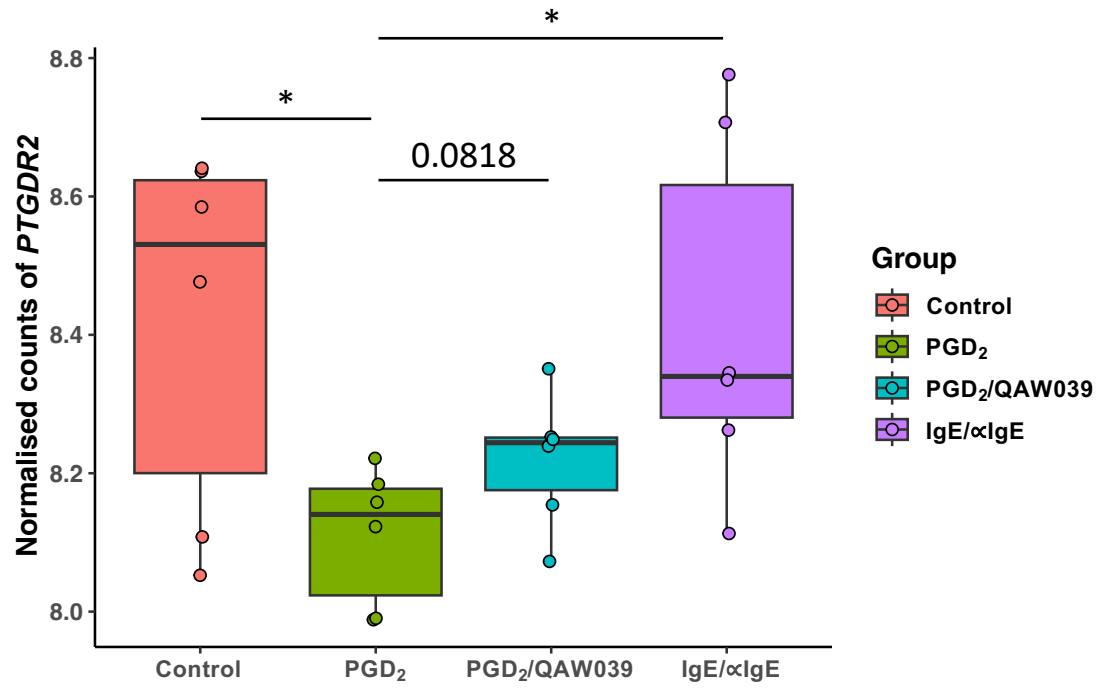

Supplementary Fig. 6 Expression levels of *PTGDR2* in basophils after different treatments detected by RNAseq. Normalised counts of *PTGDR2* were retrieved using the Variance Stabilizing Transformation (VST) method. Centre line, median; box limits, upper and lower quartiles; whiskers, 1.5x interquartile range; points, each individual replicate. **p*<0.05; n=6.

Suppl. Figure 7

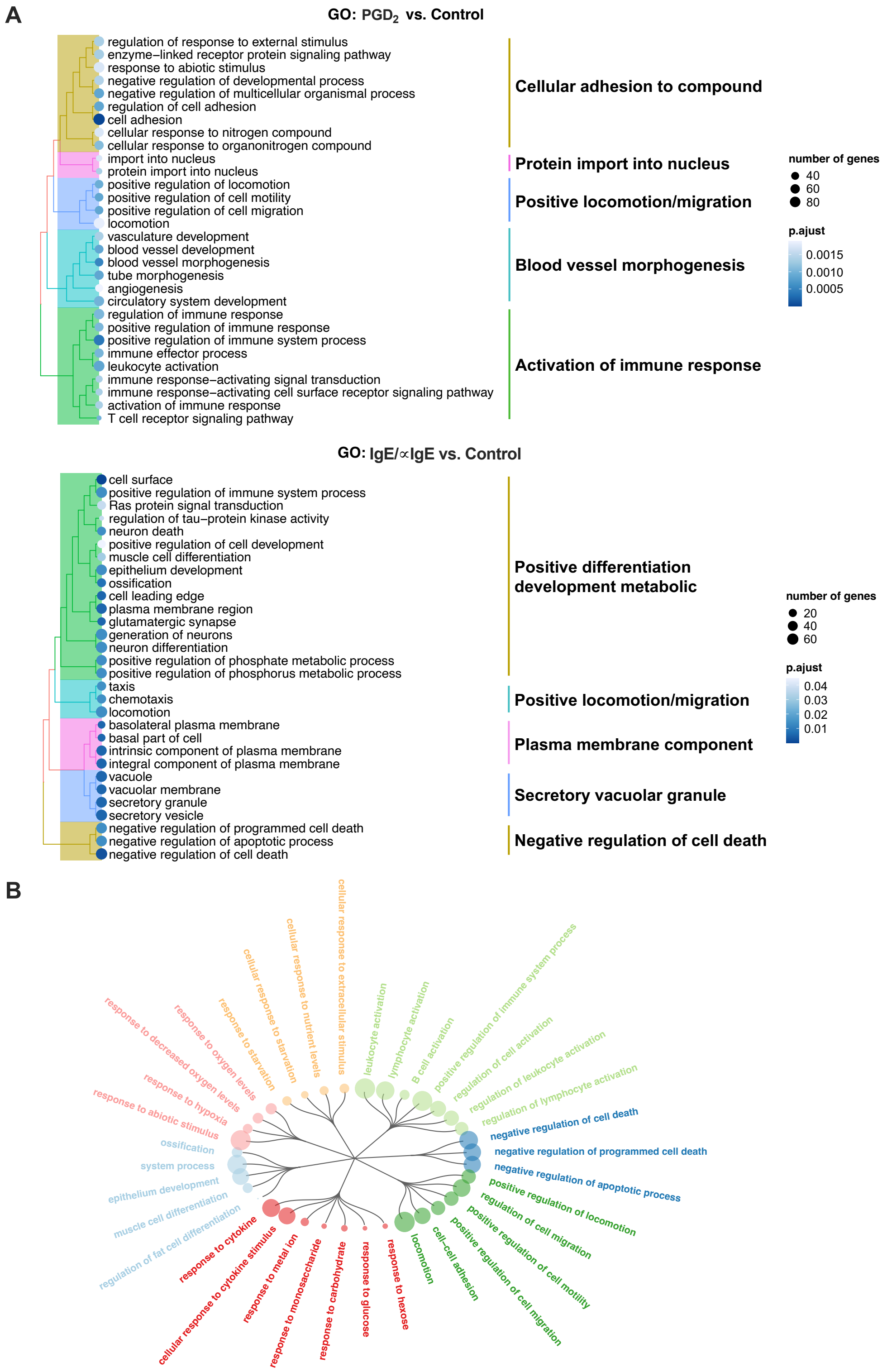

Supplementary Fig. 7 Effect of PGD_2 and IgE on the transcriptional profiles of basophils detected by RNAseq. **A** Clustering of top 30 Gene Ontology (GO) terms (biological processes, $p < 0.05$) enriched in the comparison between control and PGD_2 or IgE. **B** Shared biological functions between PGD_2 and IgE stimulations in basophils ($p < 0.05$). Biological function GO terms were retrieved from the pathway enrichment results from both PGD_2 and IgE stimulations and presented in a hierarchical dendrogram. Each colour represents a biological function, which consists of several similar GO terms.

Suppl. Figure 8

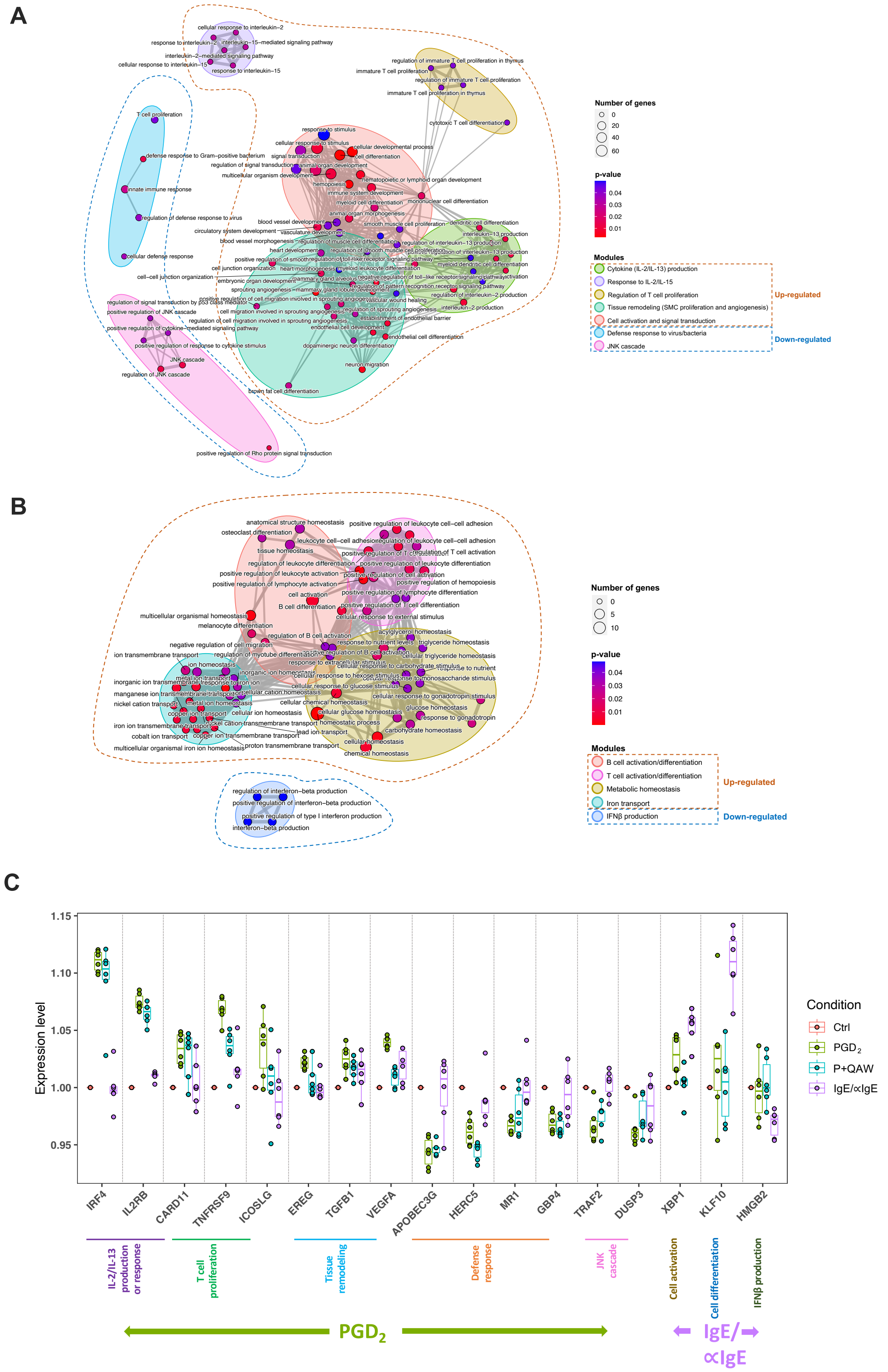

Supplementary Fig. 8 Distinct effects of PGD_2 and IgE on functional modules in basophils. **A** and **B** Function modules clustered using GO semantic similarity analysis in PGD_2- (**A**) or IgE- (**B**) treated basophils. **C** Change of key genes involved in each module among different stimulations. Expression level shown on the y-axis refers to the mean of normalized counts for each gene across all samples. n=6.

Suppl. Figure 9

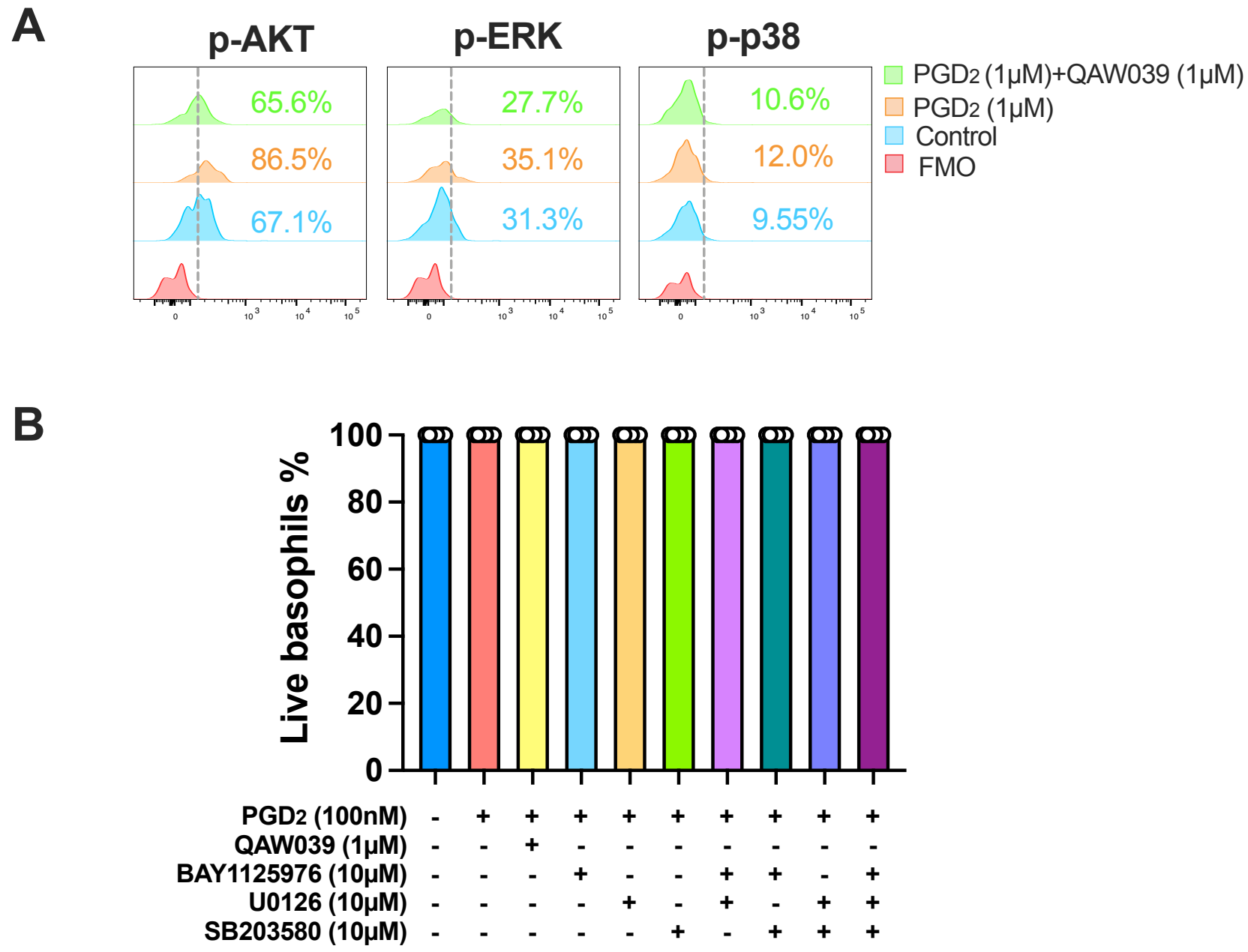

Supplementary Fig. 9 Effects of PGD_2 and various inhibitors on signal pathways and survival. **A** Representative histograms to show the frequencies of phosphoproteins (p-AKT, p-ERK and p-p38) positive cells in basophils of fresh blood were upregulated after incubation with 1 µM PGD_2 for 1 h, which were reversed by 1 µM QAW039. Fluorescence minus one (FMO) was used for gating position setting. **B** Viability of basophils after 1 h treatment with indicated inhibitors followed by 6 h PGD_2 stimulation detected with flow cytometry using Zombie Aqua. n=6.

Suppl. Figure 10

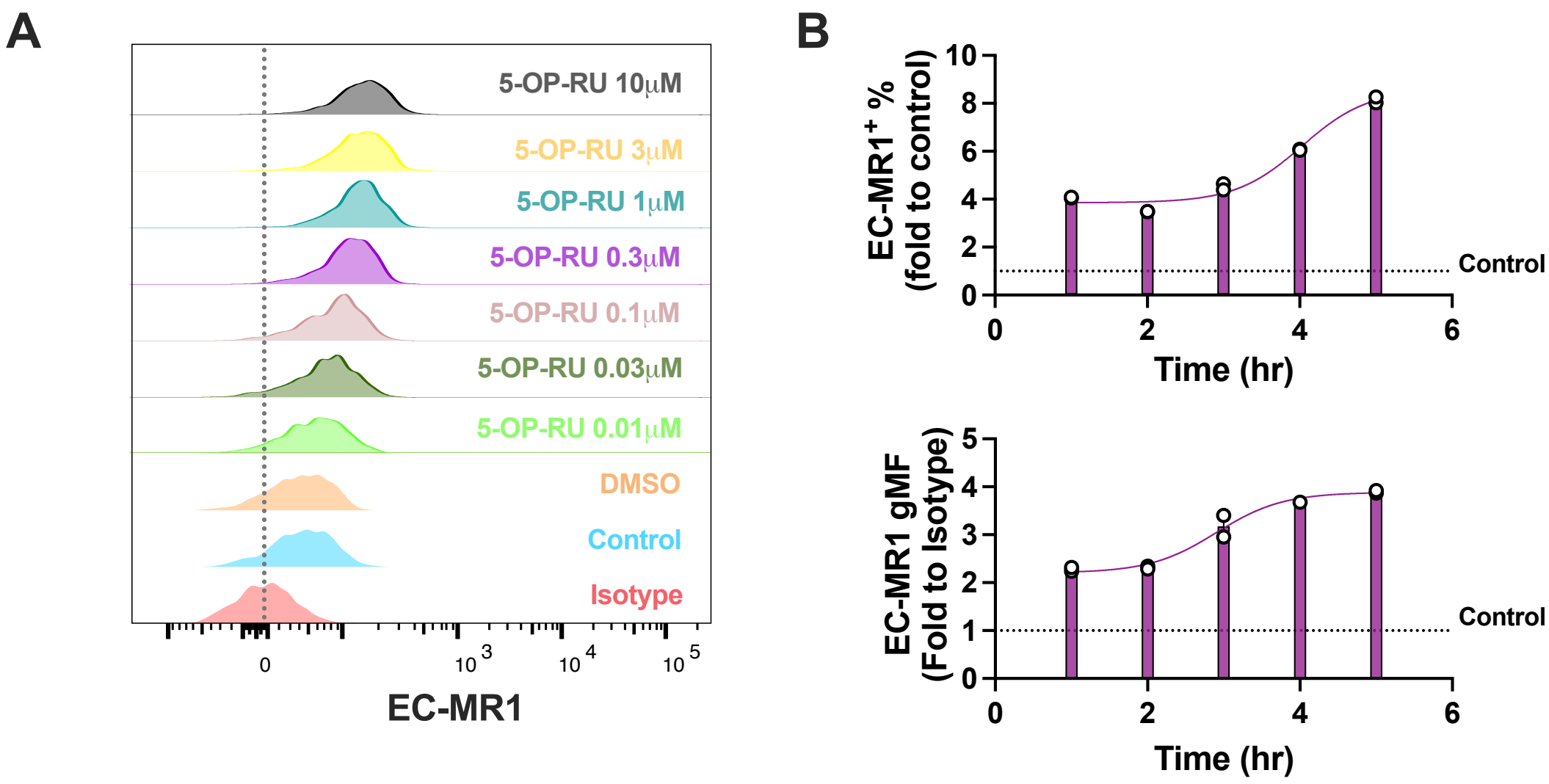

Supplementary Fig. 10 The effect of 5-OP-RU on the expression of EC-MR1 in basophils measured with flow cytometry. **A** Representative histogram of EC-MR1 on basophils stimulated by various doses of 5-OP-RU. **B** Time course of 5-OP-RU treatment on EC-MR1 expression in basophils. n=2.

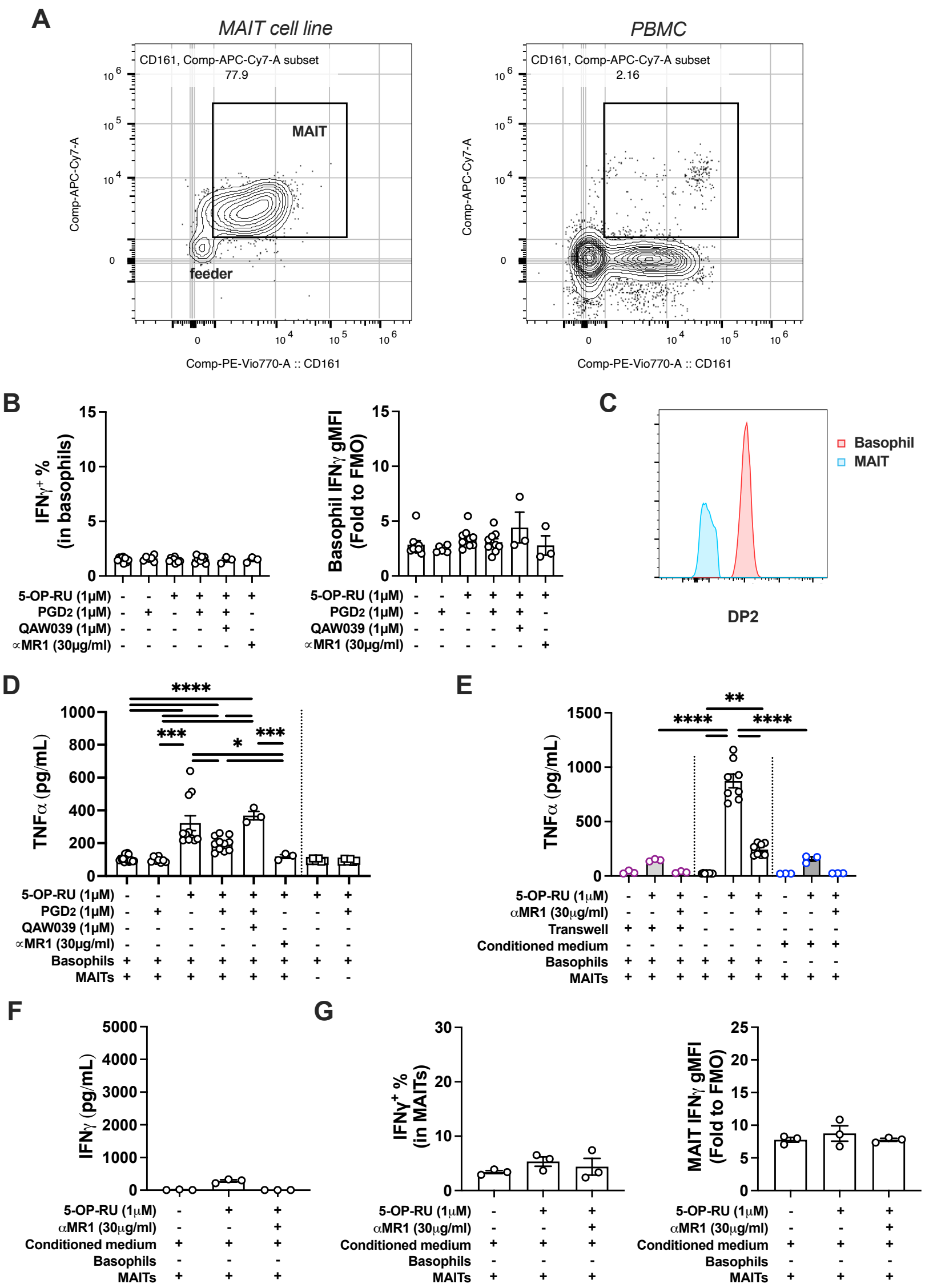

Supplementary Fig. 11 The effect of PGD_2/DP2 on MR1-mediated antigen-presenting function of basophils measured with flow cytometry or ELISA. **A** Representative plot of MAIT cell line showing MAIT cells and γ-irradiated PBMC feeders. PBMC plot is used for comparison. **B** IFN-γ+ basophils and IFN-γ levels in gated basophils in basophil/MAIT cell co-cultures in the presence or absence of 5-OP-RU, with or without PGD_2 pre-treatment with or without the addition of QAW039 in basophils. An anti-MR1 antibody was used as a control for MR1 blockade. n=9 for control, PGD_2, 5-OP-RU or 5-OP-RU/PGD_2, and n=3 for 5-OP-RU/PGD_2/QAW039 or 5-OP-RU/∝MR1. **C** Expression of DP2 in basophils and MAIT cells in the co-culture. **D** and **E** TNFα levels in the supernatants from co-cultures or basophil alone cultures (**D**), from co-cultures with transwell inserts or from MAIT cells incubated with basophil-conditioned media (**E**) were measured by ELISA. n=11 for control, PGD_2, 5-OP-RU or 5-OP-RU/PGD_2, and n=3 for 5-OP-RU/PGD_2/QAW039 or 5-OP-RU/∝MR1; n=6 for the samples without MAITs in **D**. n=3 for the samples using transwell or conditioned medium, n=8 for other samples in **E**. **F** and **G** MAIT cells treated with basophil-conditioned media. IFN-γ levels in the supernatants from conditioned medium-treated MAIT cells were measured by ELISA (**F**), and IFN-γ+ MAIT cells and IFN-γ levels in gated MAIT cells were analysed by flow cytometry (**G**). n=3. *p<0.05, **p<0.01, ***p<0.001, ****p<0.0001.

Suppl. Figure 12

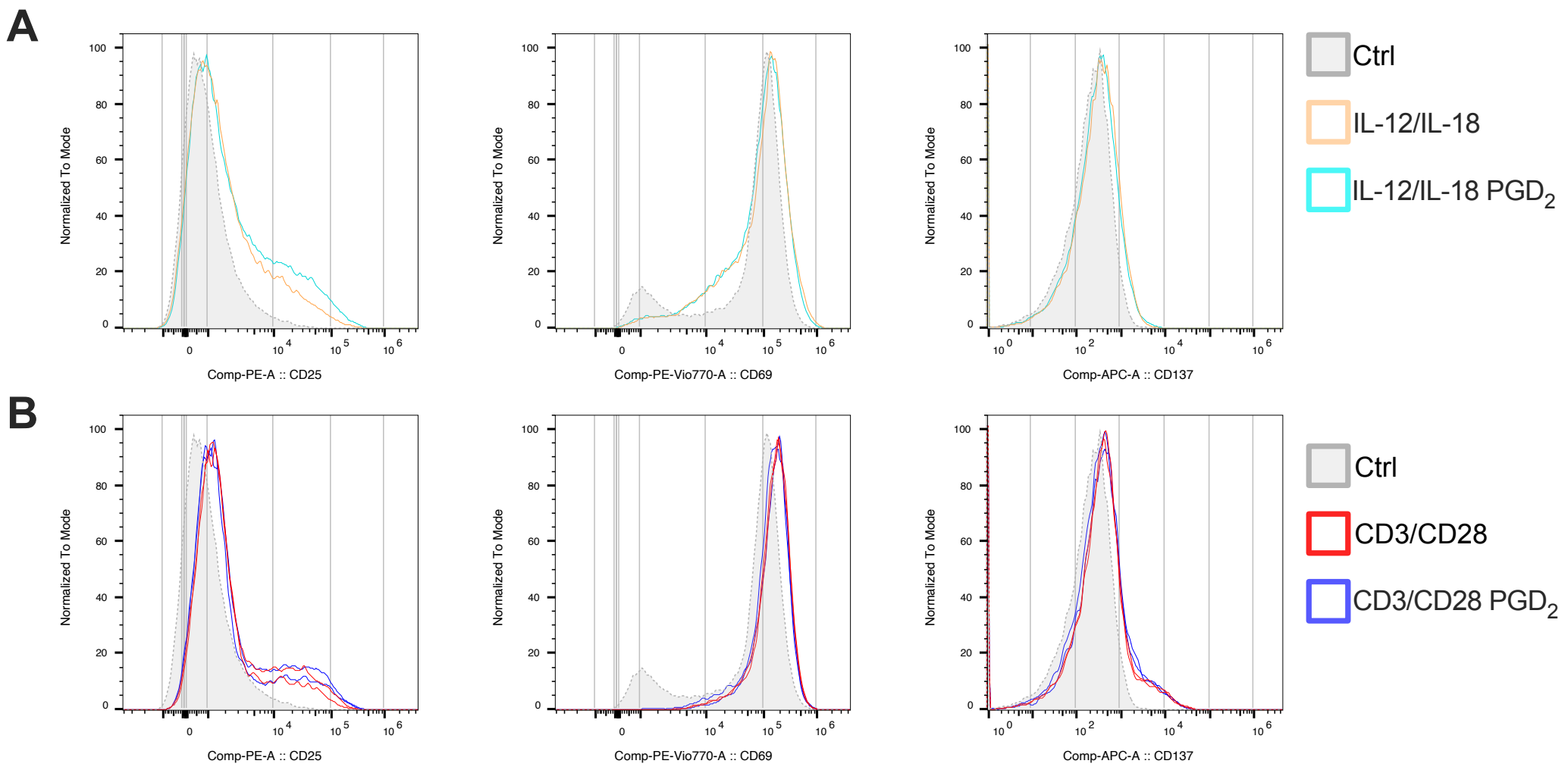

Supplementary Fig. 12 PGD_2 exerts no direct effect on MAIT cell activation. Representative histograms show the levers of activation markers (CD25, CD69 and CD137) in MAIT cells after stimulation with 50 ng/ml IL-12/IL-18 (**A**) or 2 µg/ml anti-CD3/CD28 (**B**) in the absence or presence of 1 µM PGD_2. n = 3 independent experiments.

Suppl. Figure 13

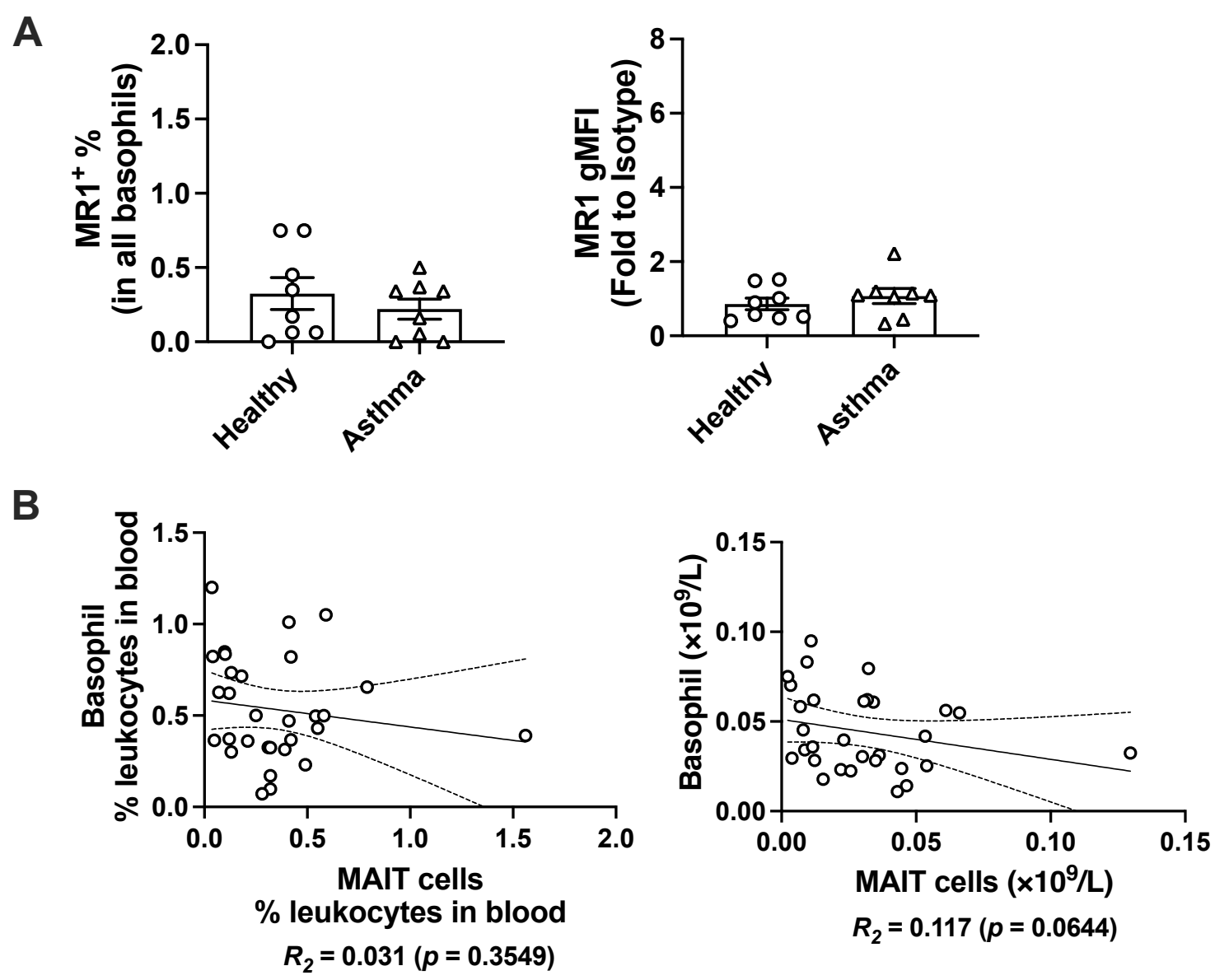

Supplementary Fig. 13 Basophil MR1 and MAIT cells in blood samples. **A** Comparison of EC-MR1 expression in blood basophils between healthy individuals and patients with asthma. n=8. **B** Correlation analysis between basophil frequencies or counts and MAIT cell frequencies or counts in peripheral blood. n=30.
