# Supplementary material for: The PGD2/DP2 axis promotes pro-inflammatory responses but attenuates an antigen-presenting role of human basophils
Source: Commun Biol. 2026 May 20;9:1079. doi: 10.1038/s42003-026-10286-w (PMC13457600; doi:10.1038/s42003-026-10286-w)
Supplement: Supplementary file 2 [file 42003_2026_10286_MOESM2_ESM.pdf]

nature portfolio

Corresponding author(s): Luzheng Xue & Jian Luo

Last updated by author(s): Apr 20, 2026

Reporting Summary

Nature Portfolio wishes to improve the reproducibility of the work that we publish. This form provides structure for consistency and transparency in reporting. For further information on Nature Portfolio policies, see our Editorial Policies and the Editorial Policy Checklist.

Statistics

For all statistical analyses, confirm that the following items are present in the figure legend, table legend, main text, or Methods section.

n/a	Confirmed	
☐	☒	The exact sample size (*n*) for each experimental group/condition, given as a discrete number and unit of measurement
☐	☒	A statement on whether measurements were taken from distinct samples or whether the same sample was measured repeatedly
☐	☒	The statistical test(s) used AND whether they are one- or two-sided<br>*Only common tests should be described solely by name; describe more complex techniques in the Methods section.*
☒	☐	A description of all covariates tested
☐	☒	A description of any assumptions or corrections, such as tests of normality and adjustment for multiple comparisons
☐	☒	A full description of the statistical parameters including central tendency (e.g. means) or other basic estimates (e.g. regression coefficient) AND variation (e.g. standard deviation) or associated estimates of uncertainty (e.g. confidence intervals)
☐	☒	For null hypothesis testing, the test statistic (e.g. *F*, *t*, *r*) with confidence intervals, effect sizes, degrees of freedom and *P* value noted<br>*Give P values as exact values whenever suitable.*
☒	☐	For Bayesian analysis, information on the choice of priors and Markov chain Monte Carlo settings
☒	☐	For hierarchical and complex designs, identification of the appropriate level for tests and full reporting of outcomes
☐	☒	Estimates of effect sizes (e.g. Cohen's *d*, Pearson's *r*), indicating how they were calculated

Our web collection on statistics for biologists contains articles on many of the points above.

Software and code

Policy information about availability of computer code

Data collection	SPECTROstar 2.10; Galapagos 1.3.0.0; BD FACSDiva 8.0.1; SpectroFlo 3.3.0; Bio-Rad CFX Manager 3.1; Novaseq6000
Data analysis	FlowJo (10.8.1), Prism (10.3.1), R (4.2.2)

For manuscripts utilizing custom algorithms or software that are central to the research but not yet described in published literature, software must be made available to editors and reviewers. We strongly encourage code deposition in a community repository (e.g. GitHub). See the Nature Portfolio guidelines for submitting code & software for further information.

Data

Policy information about availability of data

All manuscripts must include a data availability statement. This statement should provide the following information, where applicable:

- Accession codes, unique identifiers, or web links for publicly available datasets
- A description of any restrictions on data availability
- For clinical datasets or third party data, please ensure that the statement adheres to our policy

The bulk-RNAseq datasets generated and/or analysed during the current study are available on the Gene Expression Omnibus (Accession ID: GSE328453).

Research involving human participants, their data, or biological material

Policy information about studies with human participants or human data. See also policy information about sex, gender (identity/presentation), and sexual orientation and race, ethnicity and racism.

Reporting on sex and gender	Findings apply to both sexes. Sex was not considered as a covariate in the study design. Sex was neither self-reported nor assigned. Information on sex was obtained from our patient database. The number of participants of each sex is summarized in Table S1.
Reporting on race, ethnicity, or other socially relevant groupings	N/A
Population characteristics	Age, Sex, Atopy, BMI
Recruitment	Patients meeting the diagnostic criteria for various asthma phenotypes (Chung et al., 2014, Eur Respir J, 43:343), along with healthy controls, were recruited.
Ethics oversight	N/A

Note that full information on the approval of the study protocol must also be provided in the manuscript.

Field-specific reporting

Please select the one below that is the best fit for your research. If you are not sure, read the appropriate sections before making your selection.

☒ Life sciences ☐ Behavioural & social sciences ☐ Ecological, evolutionary & environmental sciences

For a reference copy of the document with all sections, see nature.com/documents/nr-reporting-summary-flat.pdf

Life sciences study design

All studies must disclose on these points even when the disclosure is negative.

Sample size	The sample size was determined according to the consistency and reproducibility of the findings. Because this study was not a clinical trial, a priori sample size calculation was not performed.
Data exclusions	No data were excluded.
Replication	Appropriate replicates were conducted to ensure reproducibility, especially in cases where the p-values did not reach statistical significance.
Randomization	N/A
Blinding	N/A

Reporting for specific materials, systems and methods

We require information from authors about some types of materials, experimental systems and methods used in many studies. Here, indicate whether each material, system or method listed is relevant to your study. If you are not sure if a list item applies to your research, read the appropriate section before selecting a response.

Materials & experimental systems

n/a	Involved in the study	
☐	☒	Antibodies
☒	☐	Eukaryotic cell lines
☒	☐	Palaeontology and archaeology
☒	☐	Animals and other organisms
☐	☒	Clinical data
☒	☐	Dual use research of concern
☒	☐	Plants

Methods

n/a	Involved in the study	
☒	☐	ChIP-seq
☐	☒	Flow cytometry
☒	☐	MRI-based neuroimaging

Antibodies

Antibodies used	CD11b-PE (clone ICRF44) Cat. 12-0118-41 eBioscience; CD11b-FITC (clone CBRM1/5) Cat. 301403 BioLegend; CD14-PE Texas-Red

Antibodies used	(clone TuK4) Cat. MHCD1417Life technologies; CD19-PE-Cy7 (clone SJ25C1) Cat. 25-0198-42 eBioscience; CD41-FITC (clone HIP8) Cat. 303703 BioLegend; CD63-APC (clone H5C6) Cat. 353008 BioLegend; CD123-PerCP (clone 32703) Cat. FAB301C R&D; CD193-APC (clone 5E8) Cat. 310708 BioLegend; CD203c-BV421 (clone NP4D6) Cat. 324612 BioLegend; DP2-PE (clone BM16) Cat. 130-113-600 Miltenyi Biotec; FcεRI-PerCP (clone AER-37 (CRA-1)) Cat. 334616 BioLegend; HLA-DR-PE-Cy7 (clone Tu36) Cat. 361612 BioLegend; IL-4-BV421 (clone MP4-25D2) Cat. 500826 BioLegend; IL-5-PE (clone JES1-39D10) Cat. 500904 BioLegend; IL-13-FITC (clone 85BRD) Cat. 11-7136-42 Invitrogen; p-AKT-AlexaFluor647 (clone M89-61) Cat. 560343 BD; p-ERK-AlexaFluor647 (clone 20A) Cat. 612593 BD; p-p38-AlexaFluor647 (clone 36/p38) Cat. 612595 BD; MR1-APC (clone 26.5) Cat. 361107 BioLegend; MR1-PE (clone 26.5) Cat. 361105 BioLegend; CD3-FITC (clone OKT3) Cat. 317306 BioLegend; CD69-PE (clone FN50) Cat. 310906 BioLegend; CD137-PE (clone 4B4-1) Cat. 309804 BioLegend; IFN-γ-APC (clone B27) Cat. 506524 BioLegend; CD3-Vio Bright V600 (Clone SK7) Cat.130-129-580 Miltenyi Biotec; CD161-Vio Bright FITC (Clone 191B8) Cat. 130-113-598 Miltenyi Biotec; CD69-PE Vio770 (Clone FN50) Cat. 130-112-615 Miltenyi Biotec; CD25-PE (Clone BC96) Cat. 302606 BioLegend; CD137-APC (Clone 4B4-1) Cat. 309809 BioLegend; TCR Vα7.2-APC/Cyanine7 (Clone 3C10) Cat. 351714 BioLegend; CD161-PE Vio770 (Clone 191B8) Cat. 130-113-594 Miltenyi Biotec; TCR Vα7.2-FITC (Clone 3C10) Cat. 351703 BioLegend.
Validation	Antibodies were obtained from reputable commercial sources, with validation data and supporting references provided by the manufacturers. Isotype controls were used in selected experiments.

Clinical data

Policy information about clinical studies

All manuscripts should comply with the ICMJE guidelines for publication of clinical research and a completed CONSORT checklist must be included with all submissions.

Clinical trial registration	N/A. Not a clinical trial. Data were from a local cohort (Ethic approval: South Central-Oxford B Research Ethics Committee, UK (18/SC/0361))
Study protocol	N/A
Data collection	From 2014-2025 at John Radcliffe Hospital, Oxford, UK.
Outcomes	N/A

Plants

Seed stocks	N/A
Novel plant genotypes	N/A
Authentication	N/A

Flow Cytometry

Plots

Confirm that:

☒ The axis labels state the marker and fluorochrome used (e.g. CD4-FITC).

☒ The axis scales are clearly visible. Include numbers along axes only for bottom left plot of group (a 'group' is an analysis of identical markers).

☒ All plots are contour plots with outliers or pseudocolor plots.

☒ A numerical value for number of cells or percentage (with statistics) is provided.

Methodology

Sample preparation	For extracellular staining, whole blood, sputum cell suspensions, PBMCs, or basophils isolated using microbeads were stained with extracellular antibodies (antibody panels listed in Supplementary Table S2), either with or without prior stimulation or treatment. Subsequently, whole blood samples were lysed using BD Lysing Solution, and all samples were fixed with IC Fixation Buffer before analysis by flow cytometry. For intracellular staining, cells were fixed with IC Fixation Buffer, permeabilized with Permeabilization Buffer, and then stained with intracellular antibodies (antibody panels listed in Supplementary Table S2).
Instrument	BD LSRFortessa; BD LSRII; Cytek Aurora

Software	BD FACSDiva 8.0.1 or SpectroFlo 3.3.0 for data collection. FlowJo (10.8.1) for data analysis.
Cell population abundance	Samples were completely run on flow cytometry. The cell population varies depending on the patient phenotype and treatment. For purified cells, at least 0.1 million cells were used and the purity was > 95%.
Gating strategy	FSC-A/FSC-H for singlets; Zombie Aqua to remove dead cells; CD123, CD193, DP2, HLA-DR to gate basophils; relevant marker-expression basophils were gated.

☒ Tick this box to confirm that a figure exemplifying the gating strategy is provided in the Supplementary Information.
