# Supplementary material for: The PGD2/DP2 axis promotes pro-inflammatory responses but attenuates an antigen-presenting role of human basophils
Source: Commun Biol. 2026 May 20;9:1079. doi: 10.1038/s42003-026-10286-w (PMC13457600; doi:10.1038/s42003-026-10286-w)
Supplement: Supplementary file 3 [file 42003_2026_10286_MOESM3_ESM.pdf]

nature portfolio

Peer Review File

The PGD_2/DP2 axis promotes pro-inflammatory responses but attenuates an antigen-presenting role of human basophils

Corresponding Author: Professor Luzheng Xue

This file contains all reviewer reports in order by version, followed by all author rebuttals in order by version.

Version 0:

Reviewer comments:

Reviewer #1

(Remarks to the Author)
The authors make two central claims:
1. The PGD_2-DP2 axis directly activates human basophils, and this activation can be inhibited with a clinically used DP2 antagonist. They demonstrate this using aggregation assays, Th2 cytokine output, and histamine release, all of which decrease upon DP2 inhibition. Although DP2 inhibition has shown clinical benefit in severe asthma, the specific mechanistic effects on basophils have not been well characterized.

2. The PGD_2-DP2 axis downregulates MR1 expression on basophils, thereby limiting basophil-MAIT cell interaction. The authors provide evidence that blocking DP2 restores MR1 levels and enables basophil-driven MAIT cell activation. This presents a previously unrecognized class I-like antigen-presentation-dependent crosstalk between basophils and MAIT cells.
Together, these findings suggest a mechanistic link between PGD_2 signaling, basophil activation, and basophil-MAIT cell crosstalk-driven inflammation, with relevance to the pathogenesis of severe asthma.
The claims are novel and will be of interest to immunologists and clinicians studying asthma, basophil biology, and innate-like T cell interactions.
• The specific mechanistic effect of the PGD_2-DP2 axis on basophils has not been reported in this level of detail.
• The identification of MR1 modulation on basophils and its functional impact on MAIT cells represents a new contribution to basophil immunobiology.
• The work also has potential broader relevance for other basophil-mediated inflammatory diseases, making it appealing to a wider audience.
The novelty would be even clearer if the introduction more explicitly highlighted what is already known about basophil roles in asthma or inflammation, such as clarifying it's known role in class II presentation, versus what is genuinely new here - particularly the MR1-MAIT axis, which appears to be the major conceptual advance.

A few points where additional evidence/framing would strengthen the claims

1. Clinical relevance of MR1 modulation.
The authors provide strong mechanistic data in vitro and ex vivo, and they assess DP2-dependent basophil cytokine responses in patient samples. However, MR1 expression in severe asthma patient basophils is not assessed or shown by flow cytometry. This would help readers understand at least the clinical relevance of MR1 expression on basophils, esp. because the discussion highlights the relevance of this axis.

2. Basophil-MAIT correlation in patient samples.
Figure 1 establishes a strong rationale for looking into new basophil activation mechanisms by showing that elevated basophils and eosinophils in severe asthma are independent of IgE levels. A parallel examination of MAIT cell frequency or basophil–MAIT correlations in these same samples would nicely connect the new mechanistic axis to the clinical setting.

3. Clarity in the introduction.
The introduction could more clearly articulate what aspects of basophil biology are known (IgE-dependent versus

independent forms of basophil activation, what’s known in only mice versus humans, class II presentation) versus what the authors are newly discovering (MR1-mediated MAIT engagement regulated by PGD_2-DP2).

Reviewer #2

(Remarks to the Author)
I co-reviewed this manuscript with one of the reviewers who provided the listed reports. This is part of the Communications Biology initiative to facilitate training in peer review and to provide appropriate recognition for Early Career Researchers who co-review manuscripts.

Reviewer #3

(Remarks to the Author)
In the manuscript by Luo and colleagues, next to increase of basophils numbers in allergic asthma patients, the mechanisms of basophil activation by PGD2 is examined in detail. Effects on mediator release, adhesion and migration are shown. In addition, interesting novel insight in the effects of PGD2 on basophils is found on decrease of the MHC-I-like molecule MR1. Overall, the manuscript provides new insights in basophil functions, specifically in allergic asthma and reads well. However, I have a few remarks and questions that I would like to discuss below:

1-In page 20 only panel 1 is referred for basophil purity antibodies. Please mention here Table S2, as I assume this is where panel 1 is referring to. Why was there no check for neutrophils, eosinophils and T lymphocytes in this panel? For instance, there can be hypodense neutrophils ending up in the Percoll interlayer in which you isolated the basophils. Moreover, CD193 can be on a subpopulation of T cells.

2-The increased basophil number and percentages in blood and sputum of allergics that is under control of steroids is an addition to the original findings described before (J Allergy Clin Immunol. 1976 Dec;58(6):676-82 and Int Arch Allergy Appl Immunol 1987;84:424-6). Please include these original findings in the manuscript.

3-The essay on in vitro aggregation used in this manuscript is not well established and can just as well be the result of poor condition of cells. In the reference by Nicholls dating 1974 no effect “of purified basophils” is studied. This is just aggregation of a total leukocyte population. There is a specific basophil homotypic aggregation study published in 1993, which seems more relevant to the current study (J. Immunol. 1993 Nov 1;151(9):4926-33.). In this study it is demonstrated that homotypic aggregation is β-2 integrin (CD18) dependent and not linked to degranulation, such as after IL-3. In the current manuscript it is shown that PGD2 is less potent in degranulation, but more in migration. Whereas, in Figure 2C and C increase of CD11b is shown (please refer to original findings on increase expression and functional properties (J Immunol. 1990 Sep 15;145(6):1832-7), it is important to show in the current aggregation experiments that also CD11b/CD18 is relevant by including blocking antibodies.

4-The increase in CD63 after PGD2 is minor when compared to CD203C, but in agreement with the very low histamine release. There should be more emphasis on this in the paper and that PGD2 is very poor in inducing degranulation. It is apparent that PGD2 is activating the basophils to adhere, migrate and release IL-4, IL-13 and LTC4 and not degranulate.

5- The MFI for CD63 is quite high In resting basophils, as high as the basophil marker CD203C. This can be the result of some pre-activation or some adhering platelets. In panel 1 for the purity of basophils, it is stated that CD41, binding platelets, was included. Does that implicate no platelets, but already activated basophils?

6-Page 16, the finding that basophils are unable to produce PGD2 themselves is known for quite some time (Fed Proc 1983 May 15;42(8):2504-9) and was the basis of the discovery in the 1980’s that basophils and not mast cells are active in the late-phase allergic reactions in nose, and skin (N Engl J Med. 1985 Jul 11;313(2):65-70, J Clin Invest. 1989 May;83(5):1519-26).

7-I miss in the discussion that basophils infiltrate the allergic affected tissues in the late phase allergic reactions. That is after the tissue-dwelling mast cells have been activated by allergen-IgE-FcεRI interactions in the early phase and release histamine and PGD2. Then the basophils are attracted by this PGD2 and enter the tissue and release potentially LTC4 and IL-4/IL-13.

8-minor: Page 15, what is IL-2/3?

Reviewer #4

(Remarks to the Author)
Luo et al. have provided a comprehensive assessment of human basophil responses to PGD2, a key mediator of Type 2

immune activation. The manuscript also includes valuable characterisation of basophils in the lung and sputum of asthmatic patients compared to healthy controls, including following treatment with the aIL-5 biologic, Mepolizumab. The authors highlight the ability of PGD2 to drive basophil migration and production of Type 2 cytokines, as well as changes in gene expression and activation of signaling pathways. This highlights that PGD2 is primarily a driver of basophil activation and function, rather than a stimulator of basophil degranulation. The latter part of the manuscript focuses on the impact of PGD2 on the expression of MR1 and the ability of basophils to drive responses from MAIT cells. I have some concerns around the validity of the results relating to the impact of PGD2 on basophil antigen presentation to MAIT cells.

Primary concern relating to MAIT cell activation:
It has previously been reported that PGD2 can act directly on MAIT cells to inhibit IFNg production (Mehta et al. 2023, ICB). Based on the methods, it is unclear whether the authors washed pre-treated basophil cultures prior to co-culture with MAIT cells. Please can the authors clarify this point. There may be PGD2 remaining in the media or adhered to the surface of basophils but not internalized that could directly activate CRTH2 on MAIT cells. Given the minor changes in MR1 expression on basophils in response to PGD2, the impact of PGD2-pretreatment prior to basophil-MAIT cell co-culture is very striking. I would find this data more convincing if the authors include further controls with PGD2/MAIT cells alone and MAIT-basophil co-culture with PGD2 present. A further validating control would be the use of another MR1/CRTH2 co-expressing APC co-cultured with MAIT cells in the same manner.

Version 1:

Reviewer comments:

Reviewer #1

(Remarks to the Author)
The authors responded to all the comments from the previous review and edited the manuscript accordingly.

Reviewer #2

(Remarks to the Author)
I co-reviewed this manuscript with one of the reviewers who provided the listed reports. This is part of the Communications Biology initiative to facilitate training in peer review and to provide appropriate recognition for Early Career Researchers who co-review manuscripts.

Reviewer #3

(Remarks to the Author)
The revised manuscript of Luo and colleagues has improved after taking the suggestions made by the reviewers in account. There is general remark left that I will address below.

Reading the rebuttal and adapted manuscript made me somewhat worried about the hints on antigen presentation that has been added to this version of the manuscript. In the adapted versions I fell that class I and class II antigen presentation are confused. Every nucleated cells, including basophils, will have a class I presentation machinery, but only antigen-presenting cells have the capability to present on class II. For human basophils there is no indication that basophils have the internal machinery to process proteins. The current manuscript is focusing on class-I like molecules and should therefore focus on this and not mix this with class-II mechanisms, or even link this to mouse basophils. For Basophil Activation Testing (BAT) in diagnostics the lack of class II on basophils, HLA-DR, is actually one of the markers used to discriminate basophils from dendritic cells and monocytes.

I have not further comments with regards to my remarks as reviewer 3.

Reviewer #4

(Remarks to the Author)
I appreciate the additional controls provided by the authors for the co-culture experiments and for clarification of their findings compared to the published literature. I congratulate the authors on their study and its positive impact on the field of basophil biology in the lung. It is certainly interesting to speculate whether the switch in basophil-MAIT cell interactions mediated by PGD2 has an impact on MAIT cell populations in the asthmatic lung, given the reported decrease in these cells in this setting, and the outcome on disease severity.

Open Access This Peer Review File is licensed under a Creative Commons Attribution 4.0 International License, which permits use, sharing, adaptation, distribution and reproduction in any medium or format, as long as you give appropriate credit to the original author(s) and the source, provide a link to the Creative Commons license, and indicate if changes were made.
In cases where reviewers are anonymous, credit should be given to 'Anonymous Referee' and the source.
The images or other third party material in this Peer Review File are included in the article's Creative Commons license, unless indicated otherwise in a credit line to the material. If material is not included in the article's Creative Commons license and your intended use is not permitted by statutory regulation or exceeds the permitted use, you will need to obtain permission directly from the copyright holder.
To view a copy of this license, visit https://creativecommons.org/licenses/by/4.0/

Dr Weishan Huang
Editorial Board, *Communications Biology*

20 Feb 2026

Dear Dr. Huang,

re: The PGD_2/DP2 axis promotes pro-inflammatory responses but attenuates a novel antigen-presenting role of human basophils (COMMSBIO-25-9291)

Thank you very much for your recent email concerning our manuscript No. COMMSBIO-25-9291 entitled “The PGD_2/DP2 axis promotes pro-inflammatory responses but attenuates a novel antigen-presenting role of human basophils”. We found the reviewers’ comments very helpful and have addressed their comments comprehensively as detailed below.

Responses to the comments

Reviewer #1:

The authors make two central claims:
1. The PGD_2-DP2 axis directly activates human basophils, and this activation can be inhibited with a clinically used DP2 antagonist. They demonstrate this using aggregation assays, Th2 cytokine output, and histamine release, all of which decrease upon DP2 inhibition. Although DP2 inhibition has shown clinical benefit in severe asthma, the specific mechanistic effects on basophils have not been well characterized.
2. The PGD_2-DP2 axis downregulates MR1 expression on basophils, thereby limiting basophil-MAIT cell interaction. The authors provide evidence that blocking DP2 restores MR1 levels and enables basophil-driven MAIT cell activation. This presents a previously unrecognized class I-like antigen-presentation-dependent crosstalk between basophils and MAIT cells.
Together, these findings suggest a mechanistic link between PGD_2 signaling, basophil activation, and basophil-MAIT cell crosstalk-driven inflammation, with relevance to the pathogenesis of severe asthma.
The claims are novel and will be of interest to immunologists and clinicians studying asthma, basophil biology, and innate-like T cell interactions.
• The specific mechanistic effect of the PGD_2-DP2 axis on basophils has not been reported in this level of detail.
• The identification of MR1 modulation on basophils and its functional impact on MAIT cells represents a new contribution to basophil immunobiology.
• The work also has potential broader relevance for other basophil-mediated inflammatory diseases, making it appealing to a wider audience.
The novelty would be even clearer if the introduction more explicitly highlighted what is already known about basophil roles in asthma or inflammation, such as clarifying it’s known role in class II presentation, versus what is genuinely new here - particularly the MR1-MAIT axis, which appears to be the major conceptual advance.

Response – We thank the reviewer's positive comments on our manuscript. The suggestion in the last paragraph will be addressed in the following comment 3.

A few points where additional evidence/framing would strengthen the claims

1. ***Clinical relevance of MR1 modulation.***

The authors provide strong mechanistic data in vitro and ex vivo, and they assess DP2-dependent basophil cytokine responses in patient samples. However, MR1 expression in severe asthma patient basophils is not assessed or shown by flow cytometry. This would help readers understand at least the clinical relevance of MR1 expression on basophils, esp. because the discussion highlights the relevance of this axis.

Response – This is an interesting suggestion. However, as demonstrated in the manuscript, the level of MR1 on the basophil surface (EC-MR1) is very low without antigen stimulation. Therefore, it would be difficult to accurately detect the differences in EC-MR1 expression on basophils *in vivo* using untreated fresh blood samples for comparison. In response to this comment, we have compared MR1 expression in blood basophils from healthy individuals and asthmatic patients, and the results have been added in the revised manuscript.

- Page 13, line 5 from bottom (Results): A new paragraph "In addition, we examined basophil MR1 expression and MAIT cell profiles in human asthma (Fig. S13). No significant difference in EC-MR1 expression on blood basophils was observed between healthy and asthmatic individuals (Fig. S13A)……" has been added.
- Fig. S13 (Supplementary): New graphs Fig. S13A have been added.

2. Basophil-MAIT correlation in patient samples.

Figure 1 establishes a strong rationale for looking into new basophil activation mechanisms by showing that elevated basophils and eosinophils in severe asthma are independent of IgE levels. A parallel examination of MAIT cell frequency or basophil–MAIT correlations in these same samples would nicely connect the new mechanistic axis to the clinical setting.

Response – We appreciate this valuable comment. In response, additional experiments were conducted to measure MAIT cell and basophil levels in the same patient blood samples. The results revealed no significant correlation between basophils and MAIT cells, and these findings have now been included in the revised manuscript.

- Page 13, line 3 from bottom (Results): "……Moreover, there was no correlation between basophil frequencies or counts and MAIT cell frequencies or counts in peripheral blood (Fig. S13B)." has been added.
- Page 1, line 8 from bottom (Supplementary Methods): A new paragraph "For MAIT cell quantification, PBMCs were labelled with antibody Panel 10 (Table S2) and fixed using IC fixation buffer (Invitrogen)." has been added.
- Fig. S13 (Supplementary): New graphs Fig. S13B have been added.

3. Clarity in the introduction.

The introduction could more clearly articulate what aspects of basophil biology are known (IgE-dependent versus independent forms of basophil activation, what's known in only mice versus humans, class II presentation) versus what the authors are newly discovering (MR1-mediated MAIT engagement regulated by PGD_2-DP2).

Response – We accept the Reviewer's suggestion, and have added some sentences in the Introduction.

- Page 4, line 13 (Introduction): Sentences "In particular, the potential roles of human basophils in antigen presentation and asthma exacerbation have not been explored, although antigen-presenting capacity has been observed in mouse basophils[6-8]." have been added.
- Page 5, line 3 (Introduction): Sentences "Basophil numbers are increased in the patients with asthma or other allergic disorders[19-21], however, how PGD_2/DP2-mediated basophil activation, IgE/FcεR1-independent pathway, contributes to the pathogenesis of these diseases remain unclear." have been added.

Reviewer #2:

I co-reviewed this manuscript with one of the reviewers who provided the listed reports. This is part of the Communications Biology initiative to facilitate training in peer review and to provide appropriate recognition for Early Career Researchers who co-review manuscripts.

Reviewer #3:

In the manuscript by Luo and colleagues, next to increase of basophils numbers in allergic asthma patients, the mechanisms of basophil activation by PGD2 is examined in detail. Effects on mediator release, adhesion and migration are shown. In addition, interesting novel insight in the effects of PGD2 on basophils is found on decrease of the MHC-I-like molecule MR1. Overall, the manuscript provides new insights in basophil functions, specifically in allergic asthma and reads well. However, I have a few remarks and questions that I would like to discuss below:

Response – We really appreciate the Reviewer's positive comments and the stating that "the manuscript provided new insights in basophil function, specifically in allergic asthma and read well.".

1-In page 20 only panel 1 is referred for basophil purity antibodies. Please mention here Table S2, as I assume this is where panel 1 is referring to. Why was there no check for neutrophils, eosinophils and T lymphocytes in this panel? For instance, there can be hypodense neutrophils ending up in the Percoll interlayer in which you isolated the basophils. Moreover, CD193 can be on a subpopulation of T cells.

Response – We accept the Reviewer's suggestion, and have added "Table S2" into the sentence as recommended.

Regarding to the antibody panel 1, we apologize for the careless missing of CD3 from the Panel 1 list in Table S2, and have now corrected. Panel 1 is sufficient to clearly identify neutrophils, eosinophils, T lymphocytes and other leukocyte populations. Specifically, neutrophils ($SSC^{high}CD193^{-}DP2^{-}$) can be distinguished from basophils ($SSC^{low}CD3^{-}CD14^{-}CD19^{-}CD41^{-}D123^{high}CD193^{high}DP2^{high}$) based on SSC, CD193 and DP2 expression. Eosinophils ($SSC^{high}CD123^{low}CD193^{low}$) can be separated by SSC, while T lymphocytes ($SSC^{low}CD3^{+}CD123^{-}$) are identified by CD3 and CD123. Other leukocytes (B lymphocytes, monocytes) can be discriminated by CD14, CD19, CD123 and DP2. In addition, we performed the Rapid Diff II staining to further confirm that the isolated cells were basophils rather than neutrophils or eosinophils (Fig. S3B). To address the Reviewer's comment, we have revised Fig. S3A to clearly illustrate how these markers were used to verify the purity of basophils used in the study.

- Page 20, line 16 (Methods): "(Table S2)" has been added.
- Table S2 (Supplementary): Panel "1" has been added in the use of CD3 antibody.
- Fig.S3 (Supplementary): Fig. S3A has been revised.

2-The increased basophil number and percentages in blood and sputum of allergics that is under control of steroids is an addition to the original findings described before (J Allergy Clin Immunol. 1976 Dec;58(6):676-82 and Int Arch Allergy Appl Immunol 1987;84:424-6). Please include these original findings in the manuscript.

Response – We appreciate the Reviewer's comment that the enrichment of basophils in allergic patients has been previously reported. This point was already acknowledged in our Discussion (2nd paragraph), where we stated: "Basophils are enriched in eosinophilic asthma and correlated with eosinophil counts[17,25]. Our data confirmed this association, and further demonstrated that basophil counts increase with asthma severity.". Accordingly, two relevant references were originally cited to support this statement. In response to the Reviewer's suggestion, we have now incorporated the additional papers recommended by the Reviewer into the revised manuscript. However, due to the limitation on the total number of references permitted (we have already exceeded), we also removed the original reference 17 (and 10) to accommodate these additions.

- Page 5, line 3 (Introduction): Sentence "Basophil numbers are increased in the patients with asthma or other allergic disorders[19-21], ……" has been inserted.
- Page 27 (References): Original reference 17 has been deleted. New references 19 and 20 have been added.

3-The essay on in vitro aggregation used in this manuscript is not well established and can just as well be the result of poor condition of cells. In the reference by Nicholls dating 1974 no effect "of purified basophils" is studied. This is just aggregation of a total leukocyte population. There is a specific basophil homotypic aggregation study published in 1993, which seems more

relevant to the current study (J. Immunol. 1993 Nov 1;151(9):4926-33.). In this study it is demonstrated that homotypic aggregation is β-2 integrin (CD18) dependent and not linked to degranulation, such as after IL-3. In the current manuscript it is shown that PGD2 is less potent in degranulation, but more in migration. Whereas, in Figure 2C and C increase of CD11b is shown (please refer to original findings on increase expression and functional properties (J Immunol. 1990 Sep 15;145(6):1832-7), it is important to show in the current aggregation experiments that also CD11b/CD18 is relevant by including blocking antibodies.

Response – We thank the reviewer for the valuable and constructive comments. The aggregation assay shown in Fig. 2A was performed as part of other functional assays and was conducted simultaneously with other assay experiments in this study. The results indicated that aggregated basophils retained a normal capacity to produce cytokines and LTC_4 and release histamine. Therefore, it is unlikely that poor cell condition was responsible for the observed aggregation. In addition, we did not detect any difference on cell survival between aggregated and non-aggregated basophils (Fig. S9B). With regard to the reference on basophil aggregation, we agree with the Reviewer that the study by Knol et al (J. Immunol. 1993,151(9):4926), which described aggregation of purified human basophils, is more relevant to our work, although the experimental approach differs from ours. Accordingly, we have replaced the original reference (Nicholls et al.) with the paper by Knol et al as reference 22.

We would also like to note that the aggregation data represent a minor component of the current manuscript and are not a central focus of the study. Multiple adhesion molecules may potentially contribute to PGD_2-induced aggregation. In addition to CD18, as mentioned by the Reviewer, our previous study (JACI, 2015, 135:1358) suggested that CD31 and CD54 may also be involved in this process. However, due to the word limitation imposed by the journal, a detailed investigation of the mechanisms underlying basophil aggregation is beyond the scope of the present manuscript. We fully agree that this is an interesting topic. Indeed, not only the mechanisms of aggregation but also several aspects of basophil activation by PGD_2 remain to be elucidated, and we will address these questions in future studies.

- Page 27, line 8 (References): Reference 22 has been replaced with a paper by Knol et al.

4-The increase in CD63 after PGD2 is minor when compared to CD203C, but in agreement with the very low histamine release. There should be more emphasis on this in the paper and that PGD2 is very poor in inducing degranulation. It is apparent that PGD2 is activating the basophils to adhere, migrate and release IL-4, IL-13 and LTC4 and not degranulate.

Response – We accept the Reviewer's suggestion, and have added some sentences in the Results and Discussion to emphasize that PGD_2 is not a strong inducer of basophil degranulation.

- Page 8, line 1 (Results): A sentence "Notably, CD63 upregulation was less pronounced than that of CD11b and CD203c." has been inserted.

- Page 8, line 5 (Results): "……although to a lesser extent than IgE/anti-IgE stimulation, and this response" has been inserted.
- Page 16, line 14 (Discussion): A sentence "Nevertheless, the effects of PGD_2 on histamine release and CD63 upregulation are modest compared with its pronounced effects on basophil adhesion, migration, and cytokine production, indicating that PGD_2 is not a strong inducer of basophil degranulation." has been inserted.

5- The MFI for CD63 is quite high In resting basophils, as high as the basophil marker CD203C. This can be the result of some pre-activation or some adhering platelets. In panel 1 for the purity of basophils, it is stated that CD41, binding platelets, was included. Does that implicate no platelets, but already activated basophils?

Response – The levels of gMFI varies depending on the brightness of fluorochrome conjugated to the antibody, and on the flow cytometer settings used during acquisition. Therefore, gMFI values are suitable for assessing relative changes only when the same fluorophore (and same antibody) is measured under identical instrument setting. Because the antibodies against CD63 and CD203c were conjugated to different fluorochromes, their gMFI values are not directly comparable. Accordingly, CD203c gMFI should not be used to infer the level of CD63 expression.

Furthermore, the samples used for the measurement of activation markers were fresh blood, the basophils in untreated blood (control) were unlikely pre-activated.

6-Page 16, the finding that basophils are unable to produce PGD2 themselves is known for quite some time (Fed Proc 1983 May 15;42(8):2504-9) and was the basis of the discovery in the 1980's that basophils and not mast cells are active in the late-phase allergic reactions in nose, and skin (N Engl J Med. 1985 Jul 11;313(2):65-70, J Clin Invest. 1989 May;83(5):1519-26).

Response – We agree with the Reviewer that the inability of basophils to produce PGD_2 has been reported previously. We didn't claim this is a novel finding. In the Discussion (Page 16), we have clearly stated that "……<u>most studies</u> suggest that basophils produce cysLTs but not PGD2[40], although low-level of PGD_2 production by basophils has been reported[41].''. In support of this statement, we have cited two representative papers (Prostaglandins Leukot Essent Fatty Acids. 1991, 44:11-17; Am J Pathol. 2011, 179: 775-782). Due to the limitation on the number of references permitted (which we have already exceeded the limit), we were unable to include all previous reports. However, the paper by Charlesworth et al (J Clin Invest. 1989 May;83(5):1519-26) is more directly relevant to the Reviewer's comment 7 and has been cited in the revised manuscript.

- Page 28 (References): A new reference paper (37) has been added.

7-I miss in the discussion that basophils infiltrate the allergic affected tissues in the late phase allergic reactions. That is after the tissue-dwelling mast cells have been activated by allergen-IgE-FcεRI interactions in the early phase and

release histamine and PGD2. Then the basophils are attracted by this PGD2 and enter the tissue and release potentially LTC4 and IL-4/IL-13.

Response – We accept the Reviewer's suggestion, and now have added this point to the revised manuscript.

- Page 15 line 7 from bottom (Discussion): The sentences "It has been suggested that, under allergic conditions, PGD_2 is up-regulated at the early phase by activated mast cells, and that basophils are subsequently infiltrate to the affected tissue during the late phase, contributing to the second wave of histamine[37]. Our data support this hypothesis and provide further evidences demonstrating how basophils are recruited and activated by PGD2 during the late phase of diseases." have been inserted.
- Page 28 (References): A new reference paper (37) has been added.

8-minor: Page 15, what is IL-2/3?

Response – Sorry for the confusing. We have made it clear now.

- Page 15, line 13 (Discussion): "IL-2/3" has been changed to "IL-2, IL-3".

Reviewer #4:

Luo et al. have provided a comprehensive assessment of human basophil responses to PGD2, a key mediator of Type 2 immune activation. The manuscript also includes valuable characterisation of basophils in the lung and sputum of asthmatic patients compared to healthy controls, including following treatment with the aIL-5 biologic, Mepolizumab. The authors highlight the ability of PGD2 to drive basophil migration and production of Type 2 cytokines, as well as changes in gene expression and activation of signaling pathways. This highlights that PGD2 is primarily a driver of basophil activation and function, rather than a stimulator of basophil degranulation. The latter part of the manuscript focuses on the impact of PGD2 on the expression of MR1 and the ability of basophils to drive responses from MAIT cells. I have some concerns around the validity of the results relating to the impact of PGD2 on basophil antigen presentation to MAIT cells.

Response – We really appreciate that the Reviewer highlighted the discoveries delivered in this manuscript.

Primary concern relating to MAIT cell activation:
It has previously been reported that PGD2 can act directly on MAIT cells to inhibit IFNg production (Mehta et al. 2023, ICB). Based on the methods, it is unclear whether the authors washed pre-treated basophil cultures prior to co-culture with MAIT cells. Please can the authors clarify this point. There may be PGD2 remaining in the media or adhered to the surface of basophils but not internalized that could directly activate CRTH2 on MAIT cells. Given the minor changes in MR1 expression on basophils in response to PGD2, the impact of PGD2-pretreatment prior to basophil-MAIT cell co-culture is very striking. I

would find this data more convincing if the authors include further controls with PGD2/MAIT cells alone and MAIT-basophil co-culture with PGD2 present. A further validating control would be the use of another MR1/CRTH2 co-expressing APC co-cultured with MAIT cells in the same manner.

Response – We sincerely thank the reviewer for these thoughtful and insightful comments. In our experiments, basophils were not washed prior to co-culture with MAIT cells. However, we have demonstrated that MAIT cells do not express DP2 (Fig. S11C), indicating that a direct effect of PGD_2 on MAIT cells is unlikely. In the study by Mehta et al. (2023, ICB), the reported effect of PGD_2 on MAIT cells was not necessarily direct, as the MAIT cells were co-cultured with THP-1 cells. Importantly, PGD_2 had no effect on cytokine-stimulated MAIT cells, but only on antigen-stimulated MAIT cells, suggesting that the observed suppression was mediated indirectly through THP-1 cells. Consistent with this interpretation, the authors also demonstrated that MR1 expression on THP-1 cells was reduced by PGD_2. These findings are in agreement with our own results showing that PGD_2 decreases MR1 expression on basophils and consequently impairs MR1-mediated antigen presentation.

Although Mehta et al. detected a suppressive effect of PGD_2 on MAIT cell activation induced in a TCR-dependent manner (anti-CD3/CD28 with cytokines), their experiments were performed using total $CD8^+$ T cells rather than purified MAIT cells. Therefore, it remains unclear whether the effect of PGD_2 was exerted directly on MAIT cells or indirectly via non-MAIT cell populations. To directly address the reviewer's concern, we conducted additional experiments in which purified MAIT cells were stimulated either in a TCR-independent manner (IL-12 and IL-18) or in a TCR-dependent manner (anti-CD3/CD28) in the presence of PGD_2. These experiments revealed no detectable direct effect of PGD_2 on MAIT cell activation under either condition. Taken together, these results further support our conclusion that PGD_2 reduces MAIT cell activation indirectly by downregulating MR1 expression on basophils. The data from these additional experiments have now been included in the revised manuscript.

To date, we are not aware of any antigen-presenting cell population other than basophils that co-expresses MR1 and DP2. The identification of additional $MR1^+DP2^+$ antigen-presenting cells would be of interest and represents an important direction for our future research.

- Page 13, line 16 (Results): A new paragraph "To determine whether PGD_2 exerts a direct inhibitory effect on MAIT cells, MAIT cells were stimulated either in a TCR-independent manner (IL-12 and IL-18) or in a TCR-dependent manner (anti-CD3/CD28) in the presence of PGD_2 (Fig. S12). No detectable direct effect of PGD_2 on MAIT cell activation markers (CD25, CD69 and CD137) was observed under either condition." has been added.
- Page 21, line 11 (Methods): "or Cytek Aurora" has been inserted.
- Page 1, line 6 from bottom (Supplementary Methods): The method for PGD_2/MAIT cell direct interaction assay has been added.
- Page 3, Table S2 (Supplementary Tables): Antibody Panel 11 has been inserted.
- Fig. S12 (Supplementary Figures): New figure Fig. S12 have been added.

Other changes:

- The Fig 1B, Fig 2B and C, Fig 3C, Fig 6C and D, Fig S4 and Fig S5D have been converted to show the individual data points to comply with the editorial policies.
- To reduce the reference number, original references 10, 17 and 45 have been deleted.

We hope these changes and comments are satisfactory. Thank you for your consideration of this work.

Yours sincerely

Luzheng Xue, PhD

Dr Tobias Goris
Senior Editor, *Communications Biology*

20 April 2026

Dear Dr. Goris,

re: The PGD_2/DP2 axis promotes pro-inflammatory responses but attenuates an antigen-presenting role of human basophils (COMMSBIO-25-9291A)

Thank you very much for your recent email concerning our revised manuscript No. COMMSBIO-25-9291A entitled "The PGD_2/DP2 axis promotes pro-inflammatory responses but attenuates a novel antigen-presenting role of human basophils". We found the comments from the reviewer #3 and the editors very helpful and have addressed these comments comprehensively as detailed below.

<u>Responses to the comments</u>

Comments from Reviewer #3: Reading the rebuttal and adapted manuscript made me somewhat worried about the hints on antigen presentation that has been added to this version of the manuscript. In the adapted versions I fell that class I and class II antigen presentation are confused. Every nucleated cells, including basophils, will have a class I presentation machinery, but only antigen-presenting cells have the capability to present on class II. For human basophils there is no indication that basophils have the internal machinery to process proteins. The current manuscript is focusing on class-I like molecules and should therefore focus on this and not mix this with class-II mechanisms, or even link this to mouse basophils. For Basophil Activation Testing (BAT) in diagnostics the lack of class II on basophils, HLA-DR, is actually one of the markers used to discriminate basophils from dendritic cells and monocytes.

*****The editors agree with Reviewer #3 that the comparison between MHC-II- and MR1-mediated presentation is somewhat off. MR1 represents an MHC-I-like unconventional pathway for antigen presentation to MAIT cells. The authors may wish to revise this point in the Discussion. It is acceptable to comment on the MHC-II pathway in the context of basophils, but the MHC-I and MHC-I-like pathways should be compared and likely given higher priority than MHC-II in the discussion.***

Response – We sincerely thank the reviewer and the editors for this valuable comment and suggestion. We have revised the Discussion accordingly by removing the sentences referring to MHC-II and focusing instead on the comparison between MHC-I- and MR1-dependent pathways.

- Page 17, line 6 from bottom (Discussion): The sentence "Mouse basophils possess MHC class II-mediated antigen-presenting capacity and are required for the allergen-induced Th2 responses[6-8]. However, some reports claim that the expression of MHC class II in human basophils is insufficient to induce

antigen-specific T-cell activation, and human blood basophils lack the features of professional antigen-presenting cells[46,47].” has been deleted.

- Page 17, line 6 from bottom (Discussion): A sentence “In contrast to the classical MHC-I pathway, which presents endogenous peptides to $CD8^+$ T cells, the MR1-dependent pathway specifically presents small, non-peptide microbial metabolites to MAIT cells[46].” has been inserted.
- Page 32, line 5 from bottom (References): Original references 46 and 47 have been deleted, and a new reference 46 has been added.

Other changes:

- The title of the manuscript has been revised to avoid using the word “novel”.
- The formatting of the manuscript, figures and Supplementary Information has been revised to comply with the Final Revision Instructions.

We hope these changes and comments are satisfactory. Thank you for your consideration of this work.

Yours sincerely

Luzheng Xue, PhD
